# T-cell biomarkers improve urinary tract infection risk stratification beyond clinical characteristics after acute traumatic spinal cord injury

**DOI:** 10.1038/s41598-025-34852-0

**Published:** 2026-01-10

**Authors:** Oliver Schweizerhof, Christian Meisel, Christian Blex, Paolo Cinelli, Ralf Watzlawick, Tom Lübstorf, Laura-Christin Geurtz, Martin Kreutzträger, Elias Baumgartner, Julian Hirt, Magdalena Hoppe, Nadine Unterwalder, Uwe Kölsch, Ralf Böthig, Burkhard Domurath, Claudia Druschel, Klaus-Dieter Schaser, Andreas Niedeggen, Armin Curt, Michael G. Fehlings, Peter Vajkoczy, Axel Ekkernkamp, Thomas Liebscher, Jan M. Schwab, Marcel A. Kopp, Ulrike Grittner

**Affiliations:** 1 Institute of Biometry and Clinical Epidemiology, Charité – Universitätsmedizin Berlin, Charitéplatz 1, 10117 Berlin, Germany; 2 Berlin Institute of Health at Charité – Universitätsmedizin Berlin, Charitéplatz 1, 10117 Berlin, Germany; 3 Institute of Medical Immunology, Charité – Universitätsmedizin Berlin, Charitéplatz 1, 10117 Berlin, Germany; 4 Berlin-Brandenburg Center for Regenerative Therapies (BCRT), Charité - Universitätsmedizin Berlin, Augustenburger Platz 1, 13353 Berlin, Germany; 5 Department of Immunology, Labor Berlin – Charité Vivantes GmbH, Sylter Strasse 2, 13353 Berlin, Germany; 6 Spinal Cord Injury Research (Neuroparaplegiology), Department of Neurology and Experimental Neurology, Charité – Universitätsmedizin Berlin, Charitéplatz 1, 10117 Berlin, Germany; 7 Department of Trauma Surgery, University Hospital Zurich, University of Zurich, Raemistr. 100, 8091 Zurich, Switzerland; 8 Department of Neurosurgery, Freiburg University Medical Center, Freiburg, Germany; 9 Treatment Centre for Spinal Cord Injuries, BG Hospital Unfallkrankenhaus Berlin, Warener Straße 7, 12683 Berlin, Germany; 10 Spinal Cord Injury Center, Kliniken Beelitz, Paracelsusring 6a, 14547 Beelitz-Heilstätten, Germany; 11 Department of Pulmonology, DRK Kliniken Berlin Mitte, Berlin, Germany; 12 Department of Neuro-Urology, Centre for Spinal Cord Injuries, BG Klinikum Hamburg, Hamburg, Germany; 13 Center for Neuro-Urology, Kliniken Beelitz, Paracelsusring 6a, 14547 Beelitz-Heilstätten, Germany; 14 Medical Advisory Service Berlin-Brandenburg, Schlaatzweg 1, 14473 Potsdam, Germany; 15 Department of Orthopaedic and Trauma Surgery, Universitätsklinikum Carl-Gustav Carus, Dresden, Germany; 16 Spinal Cord Injury Center, University Hospital Balgrist, Forchstrasse 340, 8008 Zurich, Switzerland; 17 Division of Neurosurgery, Department of Surgery, University of Toronto, 149 College Street, Toronto, ON M5T 1P5 Canada; 18 Department of Neurosurgery, Charité – Universitätsmedizin Berlin, Augustenburger Platz 1, 13353 Berlin, Germany; 19 Trauma Surgery and Orthopedics Clinic, BG Hospital Unfallkrankenhaus Berlin, Warener Straße 7, 12683 Berlin, Germany; 20 Clinic for Trauma Surgery, Orthopaedics and Specialized Septic Surgery, Klinikum St. Georg gGmbH, Delitzscher Strasse 141, 04129 Leipzig, Germany; 21 Centre for Musculoskeletal Surgery, Charité – Universitätsmedizin Berlin, Charitéplatz 1, 10117 Berlin, Germany; 22 Department of Neurology, Spinal Cord Injury Section, Belford Center for Spinal Cord Injury, Departments of Neuroscience and Physical Medicine and Rehabilitation, The Neurological Institute, The Ohio State University, Wexner Medical Center, Columbus, OH USA

**Keywords:** Spinal cord injury, Urinary tract infections, Neurogenic bladder, Risk factors, Cellular immunity, T Lymphocytes, Neuroimmunology, Biomarkers, Risk factors, Neurogenic bladder, Urinary tract infection

## Abstract

Patients with traumatic spinal cord injury (SCI) frequently develop urinary tract infections (UTI) which may compromise neurological rehabilitation. We explored risk factors for UTI, including immunological biomarkers, in patients with acute traumatic SCI during the transition from acute to subacute phase. Two observational studies were analysed separately: a retrospective study (COaT-SCI, n = 296, follow-up 13 weeks), and a prospective immunological study (SCIentinel, n = 70, follow-up 10 weeks). Cox regression models were used to examine associations of first UTI with patient characteristics and longitudinal immunological parameters. Cumulative incidence of first UTI was approximately 50% at six weeks. Results indicated higher UTI risk in older patients and time-varying associations for SCI severity (ASIA impairment scale) and presence of accompanying injuries. Higher cellular immune competence, particularly CD8 + T-cell counts and higher IFN-γ/IL-4 ratio in functional T-cell assays, were associated with a lower UTI risk (HR [95% CI] per log-transformed unit: CD8 + T-cells 0.34 [0.18—0.65], IFN-γ/IL-4 ratio 0.53 [0.33–0.83]). Adding cellular immune markers improved models’ discriminative ability (weighted C-index at 21 days from 0.68 to 0.76 with CD8 + T-cells). These findings indicate UTI is facilitated by systemic immune alterations, with T-cell parameters representing promising predictive biomarkers requiring further validation before clinical implementation.

**Supplementary Information:**

The online version contains supplementary material available at 10.1038/s41598-025-34852-0.

## Introduction

Individuals with spinal cord injury (SCI) are highly susceptible to different types of infections^1^, and systemic inflammation after SCI compromises neurological restoration^2^. Infections acquired early after acute SCI are factors associated with worse long-term neurological recovery^3^, physical independence, and survival^4^. SCI-associated urinary tract infections (UTI) are the most prevalent infections affecting individuals with SCI, with incidence reports ranging from 10 to 68%^1^ and are related to worse functional recovery^5^, morbidity, and mortality^1,6^. Current efforts to identify risk factors for SCI-associated UTI have focused mainly on the rehabilitation^7^ or chronic phase^8^.

In the acute phase, individuals who develop UTI have longer lengths of stay during inpatient rehabilitation^5,9^ and achieve smaller improvements in physical function during rehabilitation^9^. Other SCI-induced complications, such as early pneumonia and pressure ulcers, that represent sources of systemic inflammation have also been associated with worse recovery and higher mortality up to ten years after SCI^4,10^. UTI increases the risk of serious urinary tract-associated complications including acute kidney injury, sepsis, and death^6,11^. Notably, symptoms of SCI-associated UTI can be less specific due to the neurogenic lower urinary tract dysfunction^12^, which complicates diagnosis and can delay the initiation of treatment. Prophylactic antibiotic treatment is not recommended as it increases the risk of antibiotic resistance^13^. Given these diagnostic challenges and UTI as a potential modifying factor of patient outcomes, risk stratification would be helpful to identify high-risk individuals early and thus inform decisions on targeted preventive interventions or immune therapies^2,14^. However, knowledge about risk factors for UTI in the acute and subacute phase after SCI remains limited. We hypothesised that immunological biomarkers may serve as indicators of UTI susceptibility after SCI for two main reasons. First, the cellular and humoral immune system is directly affected by the injury to the spinal cord^15^, which is characterised by profound alterations in immune cell composition and function^15,16^. Second, cellular immunity is essential for maintaining the mucosal barrier to prevent infections^17^.

This explorative study aims to address the knowledge gap regarding risk factors for UTI in the acute and subacute phase after SCI. We systematically examined associations of clinical patient characteristics and immunological biomarker candidates with the time to first UTI from very acute traumatic SCI up to four months after injury. Two observational study populations were analysed separately: A retrospective acute-care study based on real-world clinical data, and a prospective study providing longitudinal immunological assessment.

## Materials and methods

### Data sources

**Fig. 1:**
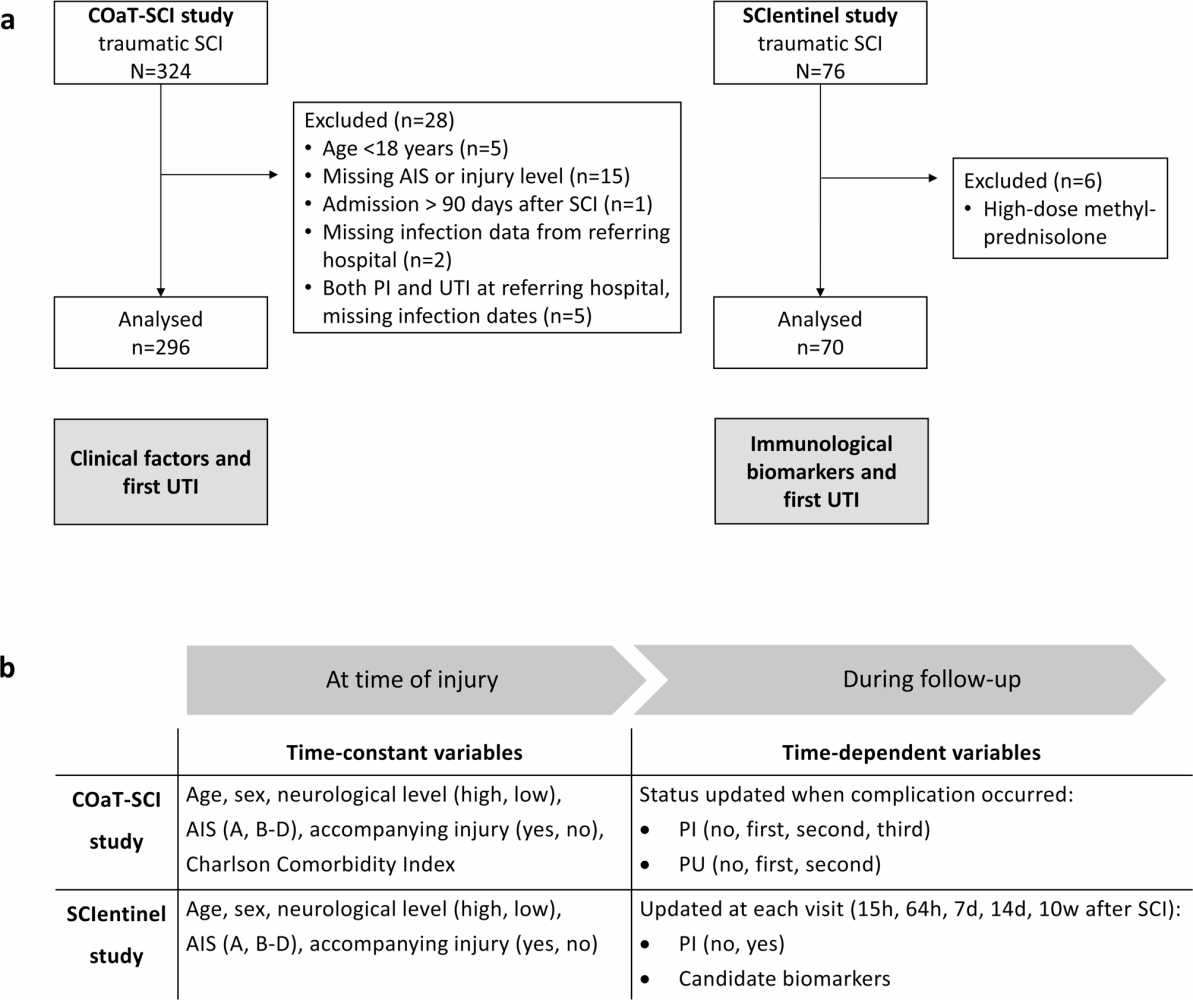
Patient selection strategy and UTI risk factor evaluation. Flow diagram **a** showing the number of patients screened and analysed in the two data sets. Each study was analysed individually. The illustration below **b** summarises which UTI risk factor information was examined over time. Abbreviations: AIS = American Spinal Injury Association Impairment Scale, PI = pulmonary infection, PU = pressure ulcer, SCI = spinal cord injury, UTI = urinary tract infection.

Two longitudinal data sources were evaluated. The single-centre Comparative Outcome and Treatment Evaluation in SCI (COaT-SCI) study conducted at the level-1 trauma centre BG Hospital Unfallkrankenhaus Berlin (ukb)^18^ and the SCI subcohort of the prospective international multicentre SCIentinel study^16^. The patient selection strategy and overview of risk factor assessment are depicted in Fig. 1. The COaT-SCI study comprises SCI-specific clinical routine and outcome data collected during primary hospitalisation after traumatic SCI. The SCIentinel study focused on repeatedly measured immunological biomarker candidates and excluded patients with immunologic confounders^16^. The studies were carried out individually, but due to the overlapping recruitment periods, 48 patients were included in both studies (16% of the COaT-SCI and 69% of the SCIentinel populations). While we acknowledge that overlap between the two study populations, it did not directly impact the analytical findings of either study because each study was conducted independently with distinct methodologies focusing on different UTI risk factors (clinical characteristics in COaT-SCI and immunological biomarkers in SCIentinel). For a more detailed data source description see Supplementary Methods and Supplementary Table S1.

### Laboratory procedures and timepoints

In the SCIentinel study, blood samples were collected at pre-specified timepoints (time windows): as early as feasible (0-31 h), 2–3 days (31-55 h), 7 days (5-9d), 14 days (11-28d), and 10 weeks (8-12w) after SCI. To reduce circadian effects, blood collections were scheduled between 7:00 and 11:00 am (except the first timepoint). Candidate biomarkers comprised differential white blood cell counts using automated hematology analysers, lymphocyte subpopulations and monocytic HLA-DR expression (mHLA-DR) measured by flow cytometry, ex vivo cytokine secretion upon mitogenic stimulation, circulating cytokines or immunotropic markers measured by immunoassays. Additionally, established ratios of leucocyte subpopulations or T-cell cytokines were investigated. Details are provided in the Supplementary Methods.

### Clinical definitions and classifications

The definitions of UTI and pulmonary infection (PI) were developed with modifications based on CDC/NHSN criteria^19^, with UTI definitions additionally incorporating SCI-specific criteria^20^. For neurological examination the International Standards for Neurological Classification of SCI were applied^21^.

#### UTI definitions

The COaT-SCI study captured symptomatic UTI that required antibiotic treatment, while in the SCIentinel study symptomatic and clinically relevant asymptomatic UTI were analysed as a composite endpoint. In the COaT-SCI study, for diagnosis of symptomatic UTI both of the following criteria applied: (a) pollakiuria, dysuria, hematuria, reduced general condition, or fever, and (b) bacteriuria (≥ 10^5^ CFU/ml urine) or initiation of specific antibiotic therapy by a physician (positive dipstick for leucocyte esterase or nitrites required). In the SCIentinel study, symptomatic UTI and clinically relevant asymptomatic UTI were captured separately but were pooled for statistical analysis. For diagnosis of clinically relevant asymptomatic UTI both of the following criteria applied: (a) bacteriuria (≥ 10^5^ CFU/ml urine) and (b) leukocyturia (≥ 100 WBC/mm^3^ urine). For diagnosis of symptomatic UTI at least one of the following additional criteria applied: fever, suprapubic or flank discomfort, bladder spasm, increased spasticity or worsening autonomic dysreflexia.

#### PI definitions

Both studies used pulmonary infection (PI) as a composite variable combining pneumonia and tracheobronchitis. In the CoAT-SCI study, PI was evaluated when clinical signs were present, including cough, fever, altered breath sounds, changes in sputum, deterioration of blood oxygen saturation, or increasing need for ventilation. When these signs occurred, chest x-ray and microbiological examination of tracheal secretions or sputum were performed. Pneumonia was defined as any clinical sign of pulmonary infection in combination with at least one radiological finding (new or changing opacities of the lung on chest x-ray). Tracheobronchitis was defined as any clinical sign of pulmonary infection combined with positive microbiologic findings while chest x-ray infiltrates were absent. For recording PI (pneumonia or tracheobronchitis), initiation of empirical or targeted antibiotic therapy was required. In the SCIentinel study, tracheobronchitis was recorded when three or more of the following criteria applied: (a) body temperature below 36.0 °C or ≥ 37.5 °C, (b) putrid secretion, (c) pathological respiration, (d) pathogenic germs in sputum, and (e) low oxygen partial pressure (< 70 mmHg) or low oxygen saturation (< 93%). Pneumonia was diagnosed if new or changing opacities on chest x-ray were present.

#### Neurological examinations

In both studies, the severity of SCI was graded at baseline using the American Spinal Injury Association Impairment Scale (AIS). Patients were categorised in the analysis as complete SCI (AIS A) vs. incomplete SCI (AIS B, C, and D). The neurological level of injury (NLI) at baseline was categorised as T4 and above (high-level) vs. T5 and below (low-level) to account for immune alterations related to disturbed signaling between the brain and the greater splanchnic nerve^22^ which originates from the spinal segments T5-T9. High-level injury results in disturbed signaling, while in low-level injuries, signaling is at least partially preserved.

The presence of accompanying injuries was dichotomised into present and not present. The definition of pressure ulcers (PU) is provided in Supplementary Table S1.

### Statistical analysis

#### General approach

Descriptive statistics for continuous variables are presented as mean (SD) or median (limits of the interquartile range, IQR). Categorical and ordinal data are reported as absolute and relative frequencies. Using Cox regression models, we examined the association between clinical patient characteristics and time to first UTI in the COaT-SCI data and explored associations between potential biomarkers and time to first UTI in the SCIentinel data. Different modelling approaches were chosen to account for time varying effects of specific covariates, for non-linear associations (i.e. for age in the COaT-SCI study), and for PI × biomarker interactions. Due to the above-mentioned study differences, the models were built differently in each study. Variables were selected based on medical background knowledge and explorative hypotheses. The timing and scope of risk factor evaluation are visualised in Fig. 1b.

#### COaT-SCI study models

Some patients transferred to the ukb study center from other hospitals after initial treatment had already developed infections at the previous facility (PI n = 16, UTI n = 4). When exact timing of infection in these cases was unavailable (PI n = 4, UTI n = 1), the infection time was set to half of the previous hospital stay. PI was handled as a time-varying covariate that changes at infection day. Over the study period the following expressions were possible “no PI”, “first PI”, “second PI”, and “third PI”. PU were handled similarly. For continuous variables, assumptions of log-linearity were checked by martingale residuals. To model a non-linear age effect, age was transformed to age squared. The proportional hazard assumptions were visually evaluated by Schoenfeld residuals. For allowing time-varying hazards, we extended the Cox model in the manner proposed by Lefebvre and Giorgi^23^ for the variables PI, AIS, and accompanying injury. For each of these three variables a function of the time-varying hazard was added. Schoenfeld residuals indicated an approximately linear time-dependent effect for covariates AIS and accompanying injury and are therefore modeled as

$$\lambda \left(t|AIS\right)={\lambda }_{0}\left(t\right) \text{exp}\left\{AIS \times ({\beta }_{AIS} + {\beta }_{AIS.t }\times t)\right\}$$

and

$$\lambda \left(t|INJ\right)={\lambda }_{0}\left(t\right) \text{exp}\left\{INJ\times ({\beta }_{INJ} + {\beta }_{INJ.t}\times t)\right\}$$

where ${\lambda }_{0}\left(t\right)$ is the baseline hazard function (the hazard when all covariates equal zero), $AIS$ is the severity of injury (complete SCI [AIS A] versus incomplete SCI [AIS BCD]) at baseline, and $INJ$ is the indicator for accompanying injury (yes vs. no) at baseline. Regression coefficients are denoted by β, with the ‘.t’ suffix indicating time-interaction terms.

We have further modified this function for PI to allow two different slopes for two time segments (piecewise linear) after exploratory analysis. One linear slope for the first 21 days and another for the time after 21 days. To determine the break of these segments, different cut-off times (from 1 to 45 days) were tested in the final model. The lowest AIC was obtained for a cut-off at 21 days and was chosen for the final model. Thus, PI was modeled as

$$\lambda \left(t|PI(t)\right)={\lambda }_{0}\left(t\right) \text{exp}\left\{PI(t) \times ({\beta }_{PI} + {\beta }_{PI.t}\times t + {\beta }_{PI.t2 }\times \left(t-21\right)I\left(t>21\right))\right\}$$

where ${\lambda }_{0}\left(t\right)$ is the baseline hazard function, $PI(t)$ is the time-dependent (time updated) categorical variable

$$PI\left(t\right)= \left\{\begin{array}{c}0\\ 1\\ 2\\ 3\end{array}\right.\begin{array}{c}\text{if } {T}_{first PI}>t \text{ or no PI occurred}\\ \text{if } {T}_{first PI}\le t \\ \text{if } {T}_{second PI}\le t \\ \text{if } {T}_{third PI}\le t\end{array},$$

and (t—21)I(t > 21) is an indicator term for estimating a different slope after day 21.

To explore possible changes in SCI treatment over the seven years of enrolment, we tested a time variable indicating the time elapsed since the first included patient, but no substantial relationship to UTI was found. Hence, the overall formula of the applied model is

$$\lambda \left( t \right) = \lambda _{0} \left( t \right)\exp\left. {\left\{ \begin{gathered} \left( {\frac{{age}}{{10}}} \right)^{2} \times \beta _{{age}} + sex \times \beta _{{sex}} + AIS \times \left( {\beta _{{AIS}} + \beta _{{AIS.t}} \times t} \right) \hfill \\ \quad + NLI \times \beta _{{NLI}} + INJ \times \left( {\beta _{{INJ}} + \beta _{{INJ.t}} \times t} \right) + PI\left( t \right) \times \left( {\beta _{{PI}} + \beta _{{PI.t}} \times t + \beta _{{PI.t2}} \times \left( {t - 21} \right)I\left( {t > 21} \right)} \right) \hfill \\ \quad + PU\left( t \right) \times \beta _{{PU}} + CCI \times \beta _{{CCI}} \hfill \\ \end{gathered} \right.} \right\}$$

where $NLI$ is the neurological level of injury (high vs. low) at baseline, $PU\left(t\right)$ is the time-dependent pressure ulcer status (similar to PI), and $CCI$ is the Charlson Comorbidity Index at baseline. Some patients (n = 15, 5.1%) died during the observation period of maximum 116d. Since death is a competing risk to first UTI, cause-specific hazard models were used. Therefore, all estimated hazard ratios (HR) of the clinical routine data represent the association with UTI risk in patients who have not yet died.

#### SCIentinel study models

Separate Cox regression models were fit for each immune parameter. Each model contained the baseline covariates sex, neurological level of injury (NLI) [high, T4 and above vs. low, T5 and below], AIS (A vs. BCD), accompanying injury (yes vs. no), age, and the time-varying covariate PI (did the patient have a PI prior/to each visit, yes vs. no) in addition to the immune parameter of interest. The SCIentinel study included two additional regression models: a basic model (as described above, but without biomarker) and an extended basic model allowing time-varying hazard for PI. A few values of laboratory measurements were below their limit of quantification (LOQ) and were replaced with LOQ/2. Laboratory measurements were transformed by using the natural logarithm (ln) and/or multiplying with a factor of 0.1 or 1000 (which is equivalent to a change in the dimension of a unit). Distributions of laboratory measurements are depicted in supplementary Fig. S3. Different types of Cox models were considered to meet the proportional hazard assumptions which were evaluated by Schoenfeld residuals: (i) stratified models to control PI by allowing different baseline hazards (ii) stratified models to control PI with additional strata(PI) × biomarker interaction (iii) interaction for PI and biomarker without stratification.

$$(i) \quad {\lambda }_{\left.(t\right)}={\lambda }_{\left.0PI(t\right)}\left(t\right) exp\left\{\left.biom(t)\times {\beta }_{biom}+ f(x)\right\}\right.$$

$$(ii) \quad {\lambda }_{\left.(t\right)}={\lambda }_{\left.0PI(t\right)}\left(t\right) exp\left\{\left.biom(t)\times {\beta }_{biom PI(t)}+ f(x)\right\}\right.$$

$${(iii) \quad \lambda }_{\left.(t\right)}={\lambda }_{0}\left(t\right) exp\left\{\left.biom(t)\times {\beta }_{biom}+PI(t)\times {\beta }_{PI}+biom(t)\times PI(t)\times {\beta }_{biomPI}+f(x)\right\}\right.$$

where ${\lambda }_{0}\left(t\right)$ is the baseline hazard function, $PI(t)$ is the time-dependent PI status

$$PI\left(t\right)= \left\{\begin{array}{c} 0 \\ 1\end{array}\right.\begin{array}{c}\text{if } {T}_{PI}>t \text{ or no PI occurred}\\ \text{if } {T}_{PI}\le t \end{array},$$

$biom(t)$ is the biomarker value at visit timepoint $t$, and

$$f\left(x\right)=\frac{age}{10}\times {\beta }_{age} + sex\times {\beta }_{sex} + AIS\times {\beta }_{AIS}+ NLI\times {\beta }_{NLI}+ INJ\times {\beta }_{INJ}$$

where the definitions were the same as described in the COaT-SCI study. In general, when a PI × biomarker interaction was present, Model (ii) was used, except for mHLA-DR where Model (iii) was applied (see also Supplementary Table S5 for model selection).

With a separate model, we evaluated how the association between PI and UTI hazard is changing over time. We extended the basic model by adding a linear function for the time-varying hazard. Therefore, in this analysis PI was modeled as follows:

$$\lambda \left(t|PI(t)\right)={\lambda }_{0}\left(t\right) exp\left\{\left.PI(t) \times ({\beta }_{PI} +{ \beta }_{PI.t }\times t)\right\}\right.$$

Since no patient died during the course of the SCIentinel study, there are no competing risks to consider.

### Sensitivity analyses

To account for differences in clinical management, we performed a sensitivity analysis for each study, adding the length of first stay in the intensive care unit (ICU) as a time-dependent covariate to the models. This was done because information on the exact method of bladder management was not available in both studies. We consider the length of first stay in the ICU as a proxy for bladder management, because all patients in the ICU generally had an indwelling catheter, while after transfer to the SCI unit, depending on the bladder function, they were switched to suprapubic catheters or intermittent catheterisation as soon as possible if sufficient voluntary voiding was not possible.

### Risk classification, missing data, and reporting standards

To assess the discriminatory ability (predictive performance) of applied Cox regression models, the concordance summary (a weighted average of the time-specific area under the receiver operating characteristic curve [AUC])^24^ at different follow-up timepoints was reported. The C-index quantifies the model’s ability to correctly rank patients by their risk, with values ranging from 0.5 (no discrimination) to 1.0 (perfect discrimination). All Cox regression models were based on complete case analysis, no imputation was performed. Due to the counting process (start-stop) format, missing biomarker values at specific time points resulted in exclusion of only those specific time points, while other available time points were retained for parameter estimation. Some patients were censored during the relevant phase of UTI occurrence (Supplementary Figure S1), mainly due to transfer to other rehabilitation centres based on capacity or proximity to home. When reporting the UTI risk, we are referring to the hazard ratios (HR) of the Cox regression models. HR are reported with two-sided 95% confidence intervals (CI). In the SCIentinel study, the Kaplan–Meier method was used to estimate the cumulative incidence of the first UTI over time. In the COaT-SCI study, the cumulative incidence function was used to account for the competing event of death. Following recommendations from the American Statistical Association^25^ and methodological experts^26,27^, our exploratory study emphasised effect size estimation with 95% CI rather than null-hypothesis significance testing. This estimation framework does not require multiple testing adjustments, which are specific to hypothesis testing. For exploratory biomarker research prioritising detection of associations, effect estimation with confidence intervals provides transparent communication of both effect magnitudes and their precision, acknowledging that independent validation is required. All analyses were conducted in R (version 4.2.2)^28^.

#### Ethics approval and adherence to guidelines

The COaT-SCI study was approved by the Institutional Review Board of Charité-Universitätsmedizin Berlin (approval number EA2/015/15). Informed consent for study participation was obtained from the participants prior to inclusion. In accordance with the regulations of the Berlin State Hospital Act, only retrospectively collected clinical care data for non-commercial research were used in cases where informed consent could not be obtained. The SCIentinel study was approved by the Ethical Committee of Charité-Universitätsmedizin Berlin (EA1/001/09), the University Health Network Research Ethics Board, Toronto (REB10-0384-AE), and the Cantonal Ethics Commission, Zurich (KEK-ZH-No. 2011–0059). All patients of the SCIentinel study were informed and gave written informed consent to participate prior to inclusion. Both studies were conducted according to the Declaration of Helsinki and GCP-principles and were reported according to the Strengthening the Reporting of Observational studies in Epidemiology (STROBE) statement^29^.

## Results

### Patient characteristics & follow-up

Patients of the COaT-SCI study (n = 296) were slightly older, more often female, less severely injured (AIS), and had more frequently a high neurological level and accompanying injuries than patients of the SCIentinel study (n = 70) (Table 1). The differences in patient characteristics reflect the different inclusion and exclusion criteria, which were more stringent in the immunological SCIentinel study to avoid additional confounding effects. The median (IQR) observation time was 13 (8, 17) weeks after SCI in the COaT-SCI and 10 (8, 10) weeks in the SCIentinel study. During these periods, UTI occurred in 218 (73.6%) and 40 (57.1%) patients, respectively. In both studies, the estimated cumulative incidence of first UTI was around 20% after three weeks and around 50% after six weeks (Table 2). In the COaT-SCI study, PI was observed more frequently (Table 2, Supplementary Fig. S1), which is in line with the higher proportion of a high neurological level, a known risk factor for PI^30,31^.

**Table 1:** Baseline demographics and clinical characteristics.

	COaT-SCI study (n = 296)	SCIentinel study (n = 70)
**Age [years]**		
Mean (SD) [range]	57.0 (18.8) [18–98]	50.8 (16.9) [22–81]
**Sex**		
Female	70 (23.4%)	13 (18.6%)
**BMI [kg/m**^**2**^**]**		
Mean (SD)	25.8 (4.4)	Not recorded
Missing	4	
**AIS at admission**		
A	131 (44.3%)	36 (51.4%)
B-D	165 (55.7%)	34 (48.6%)
**Neurological level of injury**		
High-level (C1-T4)	183 (61.8%)	29 (41.4%)
Low-level (T5-S4/5)	113 (38.2%)	41 (58.6%)
**Spine surgery**		
Yes	282 (95.3%)	70 (100%)
**High-dose methylprednisolone**		
Yes	18 (6.1%)	Exclusion criteria
**Charlson Comorbidity Index**		
No comorbidity (0)	171 (57.8%)	59 (84.3%)
Mild comorbidity (1–2)	72 (24.3%)	Not recorded
Moderate comorbidity (3–4)	27 (9.1%)	Not recorded
Severe comorbidity (> 4)	26 (8.8%)	Not recorded
**Accompanying injury**		
Yes	175 (59.3%)	32 (45.7%)
Missing	1	

Abbreviations: AIS = American Spinal Injury Association Impairment Scale, C = cervical vertebra, NLI = neurological level of injury, S = sacral vertebra, SCI = spinal cord injury, T = thoracic vertebra.

**Table 2:** Follow-up outcomes.

	COaT-SCI study (n = 296)	SCIentinel study (n = 70)
**Observation time [weeks]**		
Median (IQR)	13 (8, 16)	10 (8, 10)
**UTI**		
No	78 (26.4%)	30 (42.9%)
Yes	218 (73.6%)	40 (57.1%)
**Cumulative incidence of first UTI (95% CI)**		
Day 14	0.12 (0.08–0.16)	0.14 (0.05–0.22)
Day 21	0.20 (0.16–0.25)	0.22 (0.11–0.32)
Day 42	0.50 (0.44–0.56)	0.53 (0.38–0.64)
Day 84	0.80 (0.74–0.84)	NA
**Type of first UTI**		
Clinically relevant asymptomatic	NA	19 (47.5%)
Symptomatic	218 (100%)	21 (52.5%)
**PI frequency**		
0	146 (49.3%)	43 (61.4%)
1	84 (28.4%)	21 (30.0%)
2	42 (14.2%)	6 (8.6%)
3	24 (8.1%)	Not recorded
**Type of first PI**		
Tracheobronchitits	57 (39.3%)	19 (70.4%)
Pneumonia	88 (60.7%)	8 (29.6%)
**Pressure ulcer frequency**		
0	218 (73.6%)	51 (78.5%)
1	59 (19.9%)	14 (21.5%)
2	15 (5.1%)	Not recorded
Missing		5
**Length of first ICU stay [days]**		
Median (IQR) [range]	7 (1, 27) [0–97]	2 (0, 9) [0–65]
**Deceased within observation period**		
Yes	15 (5.1%)	0 (0%)

Abbreviations: ICU = intensive care unit, PI = pulmonary infection, UTI = urinary tract infection.

### Clinical risk factors (COaT-SCI)

**Fig. 2:**
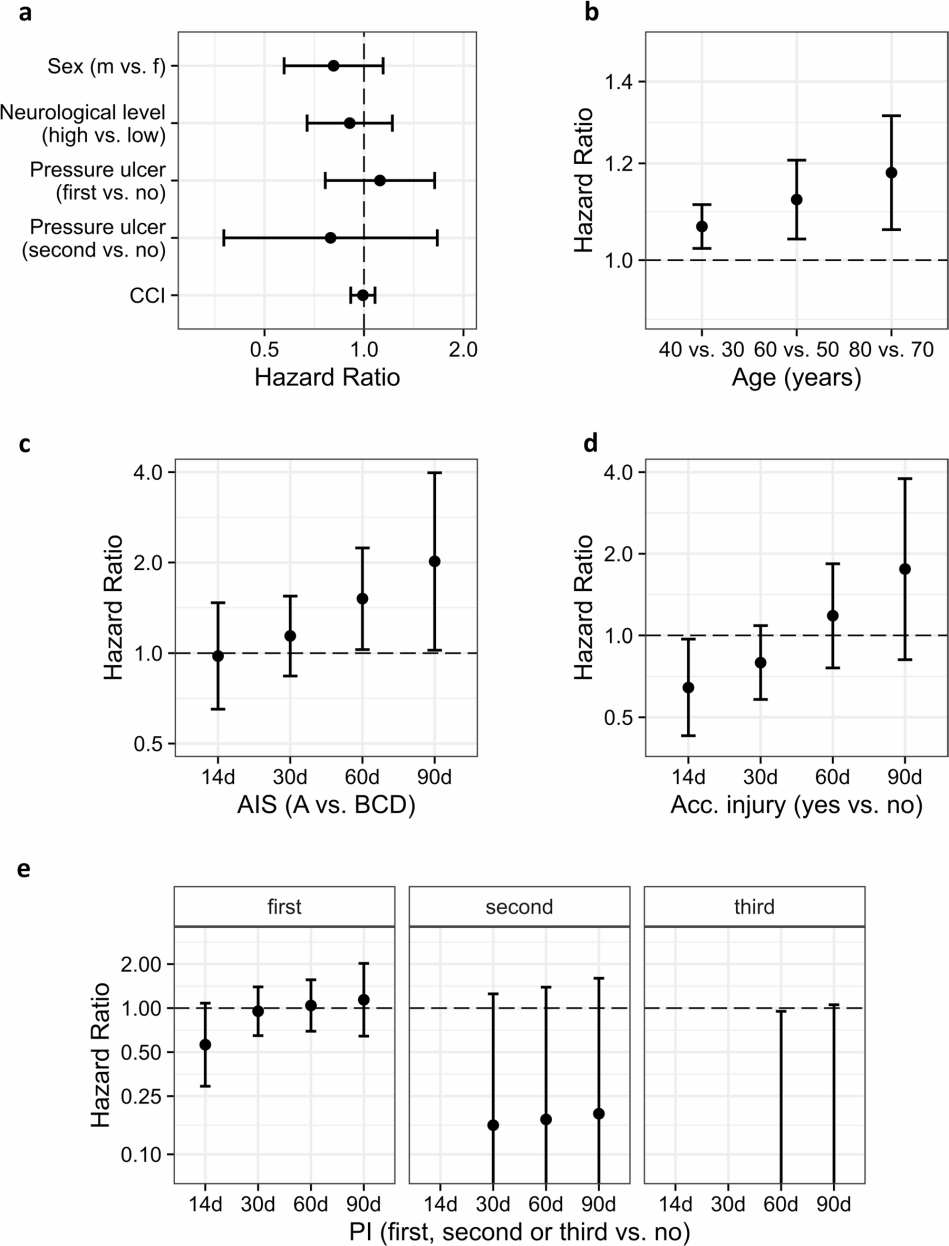
Association of clinical risk factors with first UTI in the COaT-SCI study. Hazard Ratios and 95% CI for first UTI in the COaT-SCI study for variables with constant hazard (**a**), non-linear hazard (**b**), and time varying hazard over time (**c**–**e**). All estimates were adjusted for the other shown variables in this figure (**a**–**e**). Reference categories are listed after ‘vs.’. Overall, 295 patients with SCI were included in this analysis. Abbreviations: acc. injury = accompanying injury, AIS = American Spinal Injury Association Impairment Scale, CCI = Charlson Comorbidity Index at baseline, NLI = neurological level of injury, PI = pulmonary infection, UTI = urinary tract infection.

Results of the Cox model for variables with a constant HR over time (Fig. 2a–b, Supplementary Table S2) indicate that males had a somewhat lower risk compared to females (HR [95% CI]: 0.81 [0.57—1.14]). For age there was a non-linear relationship with a general higher risk for UTI in older patients and a more pronounced age effect (steeper slope) in older age groups (Fig. 2b). In younger patients 10-years older age was related to 7% higher risk of UTI (HR [95% CI]: 1.07 [1.02–1.11], 40 versus 30 years) while in older patients the same difference was related to a 18% higher risk (HR [95% CI]: 1.18 [1.06—1.31], 80 versus 70 years). There was no substantial relationship of the NLI, PU, and Charlson Comorbidity Index (CCI) to the time to first UTI (Fig. 2, Supplementary Table S2).

Regarding factors with time-varying HR (Fig. 2c–e), no substantial association of the AIS with first UTI within one month after SCI was observed, but after two months patients graded AIS A (vs. BCD) had a higher hazard of UTI (e.g. HR [95% CI] at 60d: 1.52 [1.03–2.24]). Patients with accompanying injuries had a lower UTI risk compared to patients without accompanying injuries in the very acute phase, (e.g. HR [95% CI] at 14d: 0.64 [0.43–0.97]), but at later timepoints this association was reversed. Patients with early PI had a lower UTI risk within two weeks after SCI (e.g. HR [95% CI] at 14d: 0.52 [0.27–1.02]), and this effect diminished over time. Similarly, patients after a second or third PI had again a lower UTI risk, but here estimates were not statistically robust.

### Clinical risk factors (SCIentinel)

**Fig. 3:**
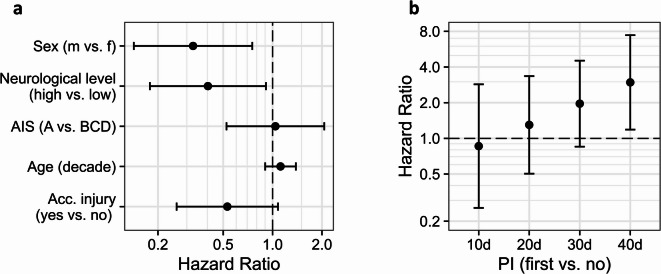
Association of clinical risk factors with first UTI in the SCIentinel study. Relative hazards (95% CI) for first UTI in the SCIentinel study using Cox regression models. Both models account for the changing relationship between PI and UTI over time: model (**a**) uses stratification by PI status, while model (**b**) directly estimates the time-varying association. Both models used demographics and clinical characteristics (age, sex, neurological level of injury, severity of injury, presence of accompanying injury, and PI). Reference categories are listed after ‘vs.’. Overall, 70 patients with SCI were included in these analyses. Abbreviations: acc. Injury = accompanying injury, AIS = American Spinal Injury Association Impairment Scale, NLI = neurological level of injury, PI = pulmonary infection, UTI = urinary tract infection.

In the Cox regression model that included only clinical parameters (basic model without immunological biomarkers), males had a reduced hazard of acquiring UTI compared to females (HR [95% CI]: 0.33 [0.15–0.71]), as did patients with high NLI (T4 and above) compared to low (T5 and below) (HR [95% CI]: 0.40 [0.18–0.91]) or patients with accompanying injury compared to patients without (HR [95% CI]: 0.53 [0.27–1.04]). Elderly patients had a slightly higher UTI risk compared to younger patients (HR [95% CI] per decade: 1.12 [0.90–1.39]), while the AIS was not substantially associated to the risk of UTI (Fig. 3a, Supplementary Table S4).

The extended basic Cox regression model was applied to evaluate the time-varying hazard for PI. Patients with PI showed a higher UTI risk at later follow-up timepoints but not at early timepoints, with HR [95% CI] ranging from 0.86 [0.26–2.86] at day ten to 2.97 [1.19–7.43] at day forty (Fig. 3B, Supplementary Table S4).

### Immunological biomarkers (SCIentinel)

**Fig. 4:**
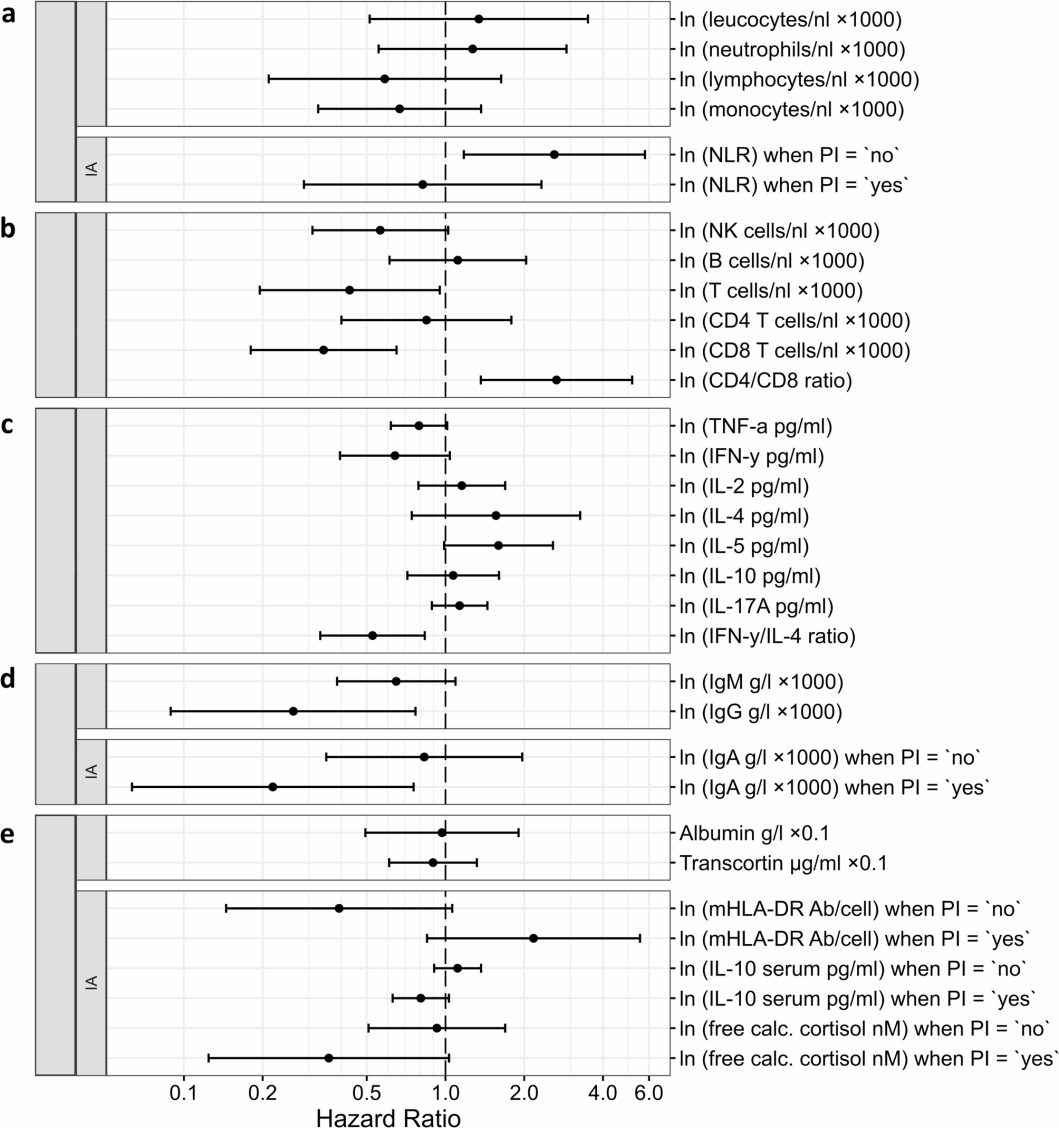
Immunological candidate markers for UTI risk in the SCIentinel study. Associations of peripheral blood immune cell counts (**a**–**b**), cytokine release of ex vivo mitogen-stimulated T cells in whole blood (**c**), serum immunoglobulin concentrations (**d**), and further immune and immune-related parameters (**e**) to first UTI using Cox regression models with time varying covariates in patients of the SCIentinel study. Subpanels are labelled IA when PI × immune parameter interaction is present, meaning the association to UTI was different after PI occurred. Shown estimates were adjusted for age, sex, neurological level of injury, severity of injury, presence of accompanying injury, and, when no PI interaction term was included, for PI. Depending on biomarker availability, 53 to 68 patients were included in these analyses. Model details, concordance summaries, patient numbers, and HR estimates are shown in Supplementary Table S5. Abbreviations: Ab = antibody, calc. = calculated, ln = natural logarithm, mHLA-DR = monocytic Human Leukocyte Antigen-DR, NLR = neutrophil to lymphocyte ratio, PI = pulmonary infection, UTI = urinary tract infection.

To explore associations of first UTI with immune parameters in the SCIentinel study, we used the basic Cox regression model, extended separately with each individual candidate biomarker as a time-varying covariate, and further modifications as appropriate (Fig. 4, Supplementary Table S5). HR of continuous variables refer to one unit change of transformed units (distributions are illustrated in Supplementary Fig. S3). Some biomarkers were differentially associated with UTI after occurrence of PI. For these, two HR were reported.

#### Immune cell quantification

Cell counts of leucocytes, neutrophils, lymphocytes, and monocytes revealed no substantial association with first UTI. A higher neutrophil-to-lymphocyte ratio (NLR) was related to a higher UTI risk in patients without previous PI (HR [95% CI]: 2.61 [1.18–5.79]), but not in patients who had already contracted PI. Considering lymphocyte subtypes, higher T-cell counts, especially cytotoxic T-cell (CD8 +) counts (HR [95% CI]: 0.43 [0.20–0.95]), and higher NK cell (CD16 +) counts (HR [95% CI]: 0.56 [0.31–1.02]) were associated with lower risk of UTI. T-helper cells (CD4 +) as well as B cells (CD19 +) were not substantially related to the hazard of UTI. The observed association of total T-cells and the CD4/CD8 ratio to UTI appears to be primarily driven by CD8 + T-cells.

#### Ex vivo cytokine release

Higher secretion of T-helper 1 (Th1) cytokines (TNF-a, IFN-γ) after in vitro T-cell stimulation was linked to a lower risk of UTI (HR [95% CI]: 0.79 [0.62–1.01], 0.64 [0.40–1.04]), whereas Th2 cytokines (IL-4, IL-5) revealed opposite associations (HR [95% CI]: 1.56 [0.74–3.27], 1.59 [0.99–2.58]). This was further supported by the association of a higher IFN-γ/IL-4 ratio with a lower UTI risk (HR [95% CI]: 0.53 [0.33–0.83]). Other associations of secreted cytokine concentrations (IL-2, IL-10, IL-17A) were not evident.

#### Peripheral blood soluble markers and mHLA-DR expression

Among serum immunoglobulin (Ig) classes, higher IgM and IgG levels were related to a lower risk of UTI (HR [95% CI]: 0.65 [0.38–1.09] and 0.26 [0.09–0.77]) but a relationship with IgA was observed only after preceding PI (HR [95% CI]: 0.20 [0.06–0.74]). Serum IL-10 and free calculated cortisol levels were associated with UTI only after PI (HR [95% CI]: 0.76 [0.61–0.94], 0.32 [0.13–0.77]). Plasma binding protein levels, albumin and transcortin, were not substantially associated with UTI. The association of higher mHLA-DR expression with risk of UTI was opposite depending on whether PI had already occurred (HR [95% CI]: 2.27 [0.90–5.75]) or not (HR [95% CI]: 0.39 [0.14–1.06]).

### Risk classification performance of Cox models

The weighted concordance index (C-index) summarises the time-specific discriminatory performance (AUC) across follow-up timepoints. The discriminatory ability of the applied Cox model in the COaT-SCI study indicated a somewhat better discrimination during the first 3 weeks after SCI than at later time points, with weighted C-indices declining from 0.66 at 14 days to 0.64, 0.61, and 0.61 at 21, 42, and 84 days, respectively (Table 3, Supplementary Table S3).

**Table 3:** Discriminatory performance of applied Cox regression models.

Model/Study	14 days	21 days	42 days	84 days
**COaT-SCI Study**				
Clinical model, n = 295	0.66	0.64	0.61	0.61
**SCIentinel Study**				
Basic model, n = 70	0.69	0.68	0.65	-
CD8 + T-cells, n = 64	0.78	0.76	0.74	-
IFN-γ/IL-4 ratio, n = 63	0.75	0.73	0.70	-
mHLA-DR, n = 64	0.77	0.76	0.74	-

Weighted concordance indices at different time points after SCI for Cox regression models analysing first UTI. The COaT-SCI study model included clinical characteristics (age, sex, NLI, AIS, accompanying injury, PI, pressure ulcer, Charlson Comorbidity Index). The SCIentinel study models included clinical variables (basic model: age, sex, NLI, AIS, accompanying injury, PI) plus one biomarker as indicated. Complete biomarker results are in Supplementary Table S5; the Incident/dynamic AUCs are shown in Supplementary Fig. S2.

Abbreviations: AIS, American Spinal Injury Association Impairment Scale; AUC, area under the receiver operating characteristic curve; mHLA-DR = monocytic Human Leukocyte Antigen-DR; NLI, neurological level of injury; PI,  pulmonary infection.

In the SCIentinel study, the discriminatory abilities of the applied Cox models declined similarly over time. The models that included biomarkers which are related to the cellular immune response (e.g. CD8 + T-cells, IFN-γ/IL-4 ratio, mHLA-DR expression) revealed better discrimination, with weighted C-indices up to 0.78 at day 14 after SCI. In comparison, the model without biomarkers had an AUC of 0.69 at day 14 (Table 3, Supplementary Table S5).

### Sensitivity analyses

The sensitivity analysis in the COaT-SCI, which additionally adjusted for length of stay in ICU as a proxy for bladder management, revealed largely similar results (Supplementary Fig. S4). The sensitivity analyses for the biomarkers (SCIentinel), that additionally included the same proxy for bladder management, largely confirmed the results of the original models. Only for two parameters with PI-interaction, i.e. NLR and mHLA-DR, the HR estimates changed slightly in patients who suffered PI prior to UTI (Supplementary Fig. S5).

## Discussion

Two observational studies were analysed to explore the associations of clinical features and immune phenotypes with the risk of first UTI in the early phase after acute traumatic SCI. The retrospective COaT-SCI study allowed investigation of relationships with clinical characteristics in greater detail, using SCI-specific clinical routine data in a larger sample. The prospective SCIentinel study, which excluded patients with immunological confounders, enabled us to explore the associations of longitudinally measured immune biomarkers with the risk of first UTI. Despite some differences in the study populations, observation time, and variable definitions, both studies that were analysed separately are reported here to provide a more comprehensive assessment of risk factors in the early phase.

The principal findings are: i) Risk estimates for injury characteristics were not constant over time and indicated that completely injured patients (AIS A) were not at higher risk of UTI in the acute phase up to 30 days after SCI, but in the subacute phase thereafter. ii) Elderly and female patients had a higher risk for UTI. iii) Markers of cellular immunity were most consistently associated with UTI. iv) Markers of humoral immunity, i.e. serum immunoglobulin levels, were related to the occurrence of UTI. v) For some biomarkers, e.g. mHLA-DR, the direction of association changed over time depending on preceding PI. vi) Considering the immunological phenotype can improve the ability to classify the UTI risk.

Previously reported demographic and clinical baseline risk factors for UTI after SCI are older age, female sex, complete SCI (AIS A)^31,32^, cervical SCI^33^ and an inability to void voluntarily during therapy^34,35^. Some of these findings are not consistent across studies, most likely due to differences in observation periods and study designs. Studies to date have largely focused on chronic SCI stages, as summarised elsewhere^36^ or on rehabilitation^34,37^. Moreover, previous studies mainly used logistic regression models or chi-square tests and thus could not acknowledge the timing and chronological order of UTI. To our knowledge, only one study focused on acute traumatic SCI and used time-to-event analysis^9^. Taking the time of UTI into account, as well as time-dependent variables that change during observation or are unknown at the start of observation, offers a methodological advantage. This enables a more differentiated investigation and helps to avoid the time-dependent bias^38^.

Providing insights across the acute and subacute phase of SCI, our results complement previous findings on associations between demographic attributes and UTI risk. Given the ageing population with SCI and the elevated risk of UTI in older age, as observed in both studies but with more certainty in the larger COaT-SCI study, these findings underscore the need to strengthen diagnostic and prevention strategies in the very acute phase of SCI. Time-varying relationships with UTI were revealed for injury characteristics in the COaT-SCI study. Here, complete SCI (AIS A vs. B-D) was linked to a higher UTI risk in the subacute but not in the acute phase after SCI. Similarly, accompanying injuries were associated with reduced UTI risk in the acute phase, but this association was reversed in the subacute phase. In the SCIentinel study, these time-varying associations were not evident, probably due to a shorter observation that reflected the acute phase of SCI more strongly. These previously unexplored associations are somewhat unexpected but have potential implications for the assessment of UTI risk during acute care of SCI patients. Differences in early clinical management for patients with more severe SCI and/or multiple trauma might explain the minor relevance of SCI severity to the UTI risk early after Injury. To address possible confounding effects of clinical management in the intensive care unit, which also includes the method of catheterisation^39,40^, sensitivity analyses were performed. Adding the length of first stay in ICU as additional covariate to the models confirmed the results of the main analyses. Therefore, we suggest that our results are largely independent of management differences related to the ICU.

Differences in treatment procedures might also be the reason why patients with high NLI (T4 and above vs. T5 and below) had a lower UTI risk very early after SCI in the SCIentinel study, whereas some previous studies from later phases of SCI reported a higher UTI risk in patients with cervical SCI^33^. PI occurs more frequently in patients with high SCI^30,31^ and very shortly after injury^16,30,41^, i.e. generally before the onset of first UTI (Supplementary Fig. S1). In both of our studies, the association between the onset of PI and first UTI varied over time. In the very early phase, patients either had a reduced UTI risk after PI, or no clear association was observed. We suggest that this time varying relationship between PI and UTI is a surrogate representing treatment differences relatable to PI that are most likely secondary effects of antibiotics^40^. Nevertheless, also some immune parameters were differentially associated with UTI when PI had occurred. Thus, also the immune response to PI may alter the susceptibility for subsequent UTI. Noteworthy, the markers with observed PI interaction are mainly markers known to be related to early infections, which are mainly PI, in patients with critical illness^42–44^ or acute SCI^41^. Among other secondary complications of SCI that may contribute to UTI susceptibility, PU were not substantially associated with UTI in the COaT-SCI study, contrary to a previous study with a different design^45^.

Among peripheral blood biomarkers, lymphocyte subpopulations revealed associations of higher cytotoxic effector cell numbers (CD8 + T-cells, NK cells) with a lower UTI risk. NK cells appear to be important for resolving UTI by reducing the bacterial loads through secretion of TNF-a^46^. The exact role of T-cells in UTI clearance is not fully understood. In our study, a higher secretion of Th1 cytokines (IFN-γ, TNF-a) after ex vivo stimulation of T-cells was related to a lower UTI risk. Complementary, a higher secretion of Th2 cytokines (IL-4, IL-5) was linked with a higher UTI risk. This opposite Th1/Th2 effects are coherent, because Th1 and Th2 responses mutually inhibit each other. A shift towards a Th2-type response after SCI is considered to attenuate neurodegenerative processes by mitigating excessive inflammation, preventing autoimmunity, and furthermore supporting neuronal regeneration or plasticity^47,48^. SCI patients with a systemic Th2 shift may benefit from such positive effects, but in turn have a reduced cell-mediated immunity. This is supported by the results of Wu et al.^49^ in a murine UTI model: After bladder infection, a Th2 directed response supports epithelial repair activities. However, this shift leads to reduced bacteria clearing Th1 response and thus contributes to recurrent UTI. The observed peripheral Th2 shift after SCI might also include T-cells in the bladder mucosa, which could explain its association with higher UTI susceptibility. Mucosal-associated invariant T-cells may be mechanistically relevant as they elicit early immune responses to uropathogenic bacteria and their dysfunctionality is associated with recurrent UTI^50^. Remarkably, diseases such as stroke share similar alterations in cellular immunity in combination with an increased UTI risk^51^, suggesting that our findings might be transferable.

Regarding humoral immunity, persistently low levels of IgG after high-level complete SCI were observed in the SCIentinel study^16^. In this subsequent analysis patients with higher IgM or IgG serum levels had a lower UTI risk, underscoring the clinical relevance of disturbed humoral immunity^16,52,53^ after SCI.

Hypercortisolism after SCI has been considered a consequence of dysregulated hypothalamic pituitary-adrenal axis (HPA-axis) and has been associated with cellular immune suppression and susceptibility for PI in experimental studies of very acute SCI^52^. Therefore, we measured free serum cortisol and its binding proteins albumin and transcortin to investigate their potential association with UTI risk. However, these markers could not be associated with the risk of UTI.

For candidate biomarkers with PI interaction, we only interpret the pre-PI estimates. An interpretation of the post-PI estimates might not be valid in this observational setting, because it is impossible to differentiate between the immunological effects of PI and effects of PI treatment. In patients with SCI, it was recently demonstrated, that higher NLR at admission are related to unfavorable AIS recovery, independent of baseline AIS^41,54^. In addition, a higher NLR was also associated with PI within the first week after SCI^41^. In the present study, a higher ratio of circulating NLR was linked to a higher risk of UTI only in patients who did not already suffered PI. Also, high mHLA-DR expression levels, that are representing an overall surrogate of cellular immune competence after trauma^55^, was related to a lower rate of first UTI in patients not yet having contracted PI. IL-10, a known candidate marker for infection risk after major surgery^44^, acts predominantly anti-inflammatory for preventing immune-mediated damage to the host^56^. We assessed IL-10 from two different sources: circulating serum IL-10 and IL-10 secretion after ex vivo T cell stimulation. Serum IL-10 reflects systemic secretion originating from various immune cell types and was more closely related to the UTI risk than IL-10 secretion after ex vivo T cell stimulation, which indicates the immunosuppressive functional state of peripheral T-cells.

Beyond individual biomarker associations, we evaluated the overall predictive potential of immune candidate biomarkers for risk stratification. The discriminatory ability was evaluated in all Cox regression models, including those with an interaction term, since the concept of confounders is not relevant for prediction purposes^57^. The concordance summaries indicated that models including parameters related to the cellular immune system provided a more accurate UTI risk allocation. Overall, the risk discrimination was better at the early time points (day 7, day 14) when UTI begin to occur. We suggest that closer monitoring of immune biomarkers could further enhance the discriminatory performance.

## Limitations

A limitation of this study is that information on treatment of PI as well as on the method of bladder voiding, which was reported to be associated with UTI risk during the acute stage^9^ and rehabilitation^34,35^, was not available. Nevertheless, in the acute phase, the care algorithm including bladder management may also depend on the patient’s age, SCI severity and level, accompanying injuries, and occurrence of PI, all of which were considered as covariates for adjustment. Furthermore, in order to account for potential additional confounding, the associations with UTI were reevaluated in a sensitivity analysis that included the length of first stay in the ICU as a surrogate for the minimum duration of indwelling catheter use. The results for the investigated clinical risk factors and for the immunological biomarker candidates were very similar in both studies. In addition, the direction of associations for our surrogate with UTI was consistent with a previous report on the duration of initial indwelling catheterisation in patients with acute traumatic SCI^9^. The use of ICU length of stay as a proxy may not fully account for individual timing of catheter management practices, which could still introduce residual confounding. In addition, due to the study design, the immunological findings refer to a SCI population without other major immune-modifying conditions or diseases, which may limit generalisability to broader SCI populations. Other possible sources of immunological variability, such as vaccination or prior infectious history, were not part of the study and could not be investigated. However, while individual immunological history may influence immune phenotypes after SCI, this does not diminish the predictive value of our measured immune parameters for UTI risk. Missing biomarker measurements at some visits could potentially introduce bias in the associations. Among patients without prior UTI and not yet censored, biomarker data completeness ranged from 81–91% at day 7, and improved to 85–94% at day 14, except for IL-17A (72–79%). Since data completeness was relatively high at the most interesting timepoint for UTI at day 14, the potential impact on our findings is likely limited. Additionally, the urinary microbiome represents another unmeasured factor that may influence immunity and UTI susceptibility. Limited knowledge exists regarding the role of the urinary microbiome in UTI pathogenesis and how it interacts with the immune system. Available evidence suggests that the urinary microbiome in neurogenic bladder patients remains similar to able-bodied controls within the first 2 months post-SCI^58^, but shifts toward unfavorable bacterial colonization in the chronic stage (> 1 year), characterised by Enterobacteriaceae enrichment and Lactobacillaceae depletion, particularly in catheterised patients^59^. This temporal pattern suggests that early UTI may also contribute to subsequent microbiome changes through immune-microbiome interactions or antibiotic effects, potentially influencing recurrent UTI patterns.

The shared subgroup is a further limitation, as it suggests that any consistency in demographic and clinical characteristics between the study populations might be due to this partial dependency rather than independent validation. However, the different research questions of the two studies ensured their specific results and conclusions remain valid and unaffected. Furthermore, the possibility of the SCIentinel study’s prospective monitoring influencing clinical behavior, a common issue in observational research, was considered, but no substantial association was found between the timing of the first UTI and the study period in the COaT-SCI cohort.

The internal consistency observed within biomarker categories (e.g., markers representing higher cellular immune competence consistently associated with lower UTI risk) and the plausibility of interaction effects (e.g., markers known to be associated with infections in critical illness showing PI interactions) support the validity of these exploratory findings, though prospective validation studies are needed.

## Conclusions

Immunological biomarkers in combination with clinical factors such as age and injury characteristics can improve UTI risk stratification after SCI, with T-cell parameters being the most promising candidates at present. Table 4 summarises the top-performing biomarkers alongside their discriminatory performance and clinical feasibility. Notably, lymphocyte subpopulations can be determined within hours in haematology or immunology laboratories, making them particularly suitable for timely risk assessment, whilst mHLA-DR quantification and especially ex vivo T-cell stimulation assays (IFN-γ/IL-4 ratio) require specialised immunology laboratories and longer turnaround times. Prospective multicentre studies are needed to establish a validated UTI risk assessment algorithm that would enable targeted preventive interventions for high-risk patients and timely UTI diagnosis through enhanced clinical surveillance during the early phase after SCI. Such an approach could potentially reduce UTI burden, prevent related renal complications, and improve rehabilitation outcomes. Therefore, disseminating knowledge on UTI risk assessment and bladder management algorithms^60^ beyond specialised SCI care centres will be essential.

**Table 4:** Biomarkers with best performance for UTI risk assessment after acute SCI.

Biomarker Model	Hazard Ratio (95% CI) [per unit]	Weighted C-index at 42 days	Turnaround Time	Laboratory Specialisation Required
CD8 + T-cells, n = 64	0.34 (0.18–0.65)	0.74	Same day (4–8 h)	Standard haematology or flow cytometer analyser
IFN-γ/IL-4 ratio, n = 63	0.53 (0.33–0.83)	0.70	> 3–5 days	Specialised immunology laboratory
mHLA-DR, n = 64	Before PI: 0.39 (0.14—1.06) After PI: 2.27 (0.90—5.75)	0.74	> 12–24 h	Flow cytometry facility

Top-performing biomarkers from the SCIentinel study with discriminatory performance and clinical feasibility. Each Cox model included age, sex, neurological level of injury, severity of injury, accompanying injury, PI, and one biomarker. Complete biomarker results are in Supplementary Table S5. Abbreviations: AIS = American Spinal Injury Association Impairment Scale, C-index = Concordance index, mHLA-DR = monocytic Human Leukocyte Antigen-DR, NLI = neurological level of injury, PI = pulmonary infection.

Moreover, treatment options for high-risk UTI patients remain limited and require further evaluation. Immune biomarkers for the individual patient’s risk of developing UTI can inform the design of interventional trials regarding inclusion criteria and patient stratification. Current research on immunomodulatory interventions in SCI primarily focuses on limiting the detrimental proinflammatory cascades and enhancing regenerative healing mechanisms^47,61,62^. Research beyond SCI is also investigating immunomodulation strategies to prevent bacterial infections. Zhang et al. reviewed the current research landscape and ongoing challenges, particularly regarding the temporal immune dynamics during bacterial infections^63^. For SCI, a nuanced understanding of spatiotemporal immune dynamics is crucial for developing therapies that enhance antibacterial immunity while preventing autoimmunity and supporting regenerative mechanisms^64^.

## Supplementary Information

Supplementary Information.

## Data Availability

Due to data protection reasons, detailed individual-level data from both studies cannot be fully shared. However, extracts of anonymised data and statistical codes can be made available by the corresponding authors upon reasonable request and signing of a data and knowledge transfer agreement.
