# Supplementary material for: T-cell biomarkers improve urinary tract infection risk stratification beyond clinical characteristics after acute traumatic spinal cord injury
Source: Sci Rep. 2026 Jan 10;16:1320. doi: 10.1038/s41598-025-34852-0 (PMC12796231; doi:10.1038/s41598-025-34852-0)
Supplement: Supplementary file 1 — Supplementary Information. [file 41598_2025_34852_MOESM1_ESM.docx]

**SUPPLEMENTAL MATERIAL**

[**Supplementary Methods** 2](#_Toc208220620)

[**Data source description** 2](#_Toc208220621)

[**Laboratory procedures** 3](#_Toc208220622)

[**Supplementary Tables** 6](#_Toc208220623)

[**Supplementary Table S1** 6](#_Toc208220624)

[**Supplementary Table S2** 8](#_Toc208220625)

[**Supplementary Table S3** 9](#_Toc208220626)

[**Supplementary Table S4** 10](#_Toc208220627)

[**Supplementary Table S5** 11](#_Toc208220628)

[**Supplementary Figures** 13](#_Toc208220629)

[**Supplementary Figure S1** 13](#_Toc208220630)

[**Supplementary Figure S2** 15](#_Toc208220631)

[**Supplementary Figure S3** 16](#_Toc208220632)

[**Supplementary Figure S4** 18](#_Toc208220633)

[**Supplementary Figure S5** 20](#_Toc208220634)

[**Supplementary References** 22](#_Toc208220635)

# **Supplementary Methods**

## **Data source description**

**Comparative Outcome and Treatment Evaluation in SCI study**

Patients with traumatic spinal cord injury (SCI) from the Comparative Outcome and Treatment Evaluation in SCI (COaT-SCI) study were enrolled in the level-1 trauma centre Unfallkrankenhaus Berlin (ukb) from October 28, 2010 to December 28, 2017. To focus on the setting of primary care and the early rehabilitation phase, we truncated the observation after day 116 (approximately 17 weeks) post SCI (see Supplementary Table S1 for details).

**SCIentinel study**

The SCIentinel study is a multicentre, prospective, longitudinal study designed to investigate associations between traumatic injury of spinal cord and neurogenic systemic immune changes.^1^ It was pre-registered at DRKS (German Clinical Trials Register; DRKS00000122; registered April 27, 2009; start date August 21, 2011). The SCI cohort of the SCIentinel study was enrolled in six clinical departments: Treatment Centre for Spinal Cord Injuries at BG Hospital Unfallkrankenhaus Berlin, Germany; Department of Neurosurgery at Charité-Universitätsmedizin Berlin, Germany; Centre for Musculoskeletal Surgery at Charité-Universitätsmedizin Berlin, Germany; Spinal Cord Injury Center, University Hospital Balgrist, University of Zurich, Switzerland; Department of Trauma Surgery, University Hospital Zurich, University of Zurich, Switzerland; Division of Neurosurgery, Department of Surgery, University of Toronto, Canada. For inclusion, patients had to be at least 18 years old with a traumatic SCI and planned surgical stabilisation or decompression. Exclusion criteria were life threating polytrauma, serious traumatic brain injury, pre-existing neoplasia, auto-immune-diseases, or chronic infections. The planned time periods for the examinations and laboratory biomarker sampling ranged from admission to the end of inpatient rehabilitation: within the first 31 hours, 31‑96 hours, 5‑9 days, 11‑28 days, and 8‑12 weeks after injury. More detailed information can be found in the SCIentinel study protocol^2^ and results publication.^1^

## **Laboratory procedures**

The blood samples used for biomarker analysis were handled under a six-digit pseudonym and the personnel running the laboratory analyses were kept blinded for the clinical characteristics of the patients. For immunophenotyping, whole blood samples were collected in Cyto-Chex® tubes (Streck, La Vista, NE, USA) enabling for preservation of morphology, antigen presenting molecules and cluster of differentiation (CD) of white blood cells for up to 14 days at room temperature, and were measured within 36 hours after blood collection. Samples for leukocyte cell differentiation and ex vivo cytokine secretion were collected using BD Vacutainer® (BD Biosciences, Heidelberg, Germany) Ethylenediaminetetraacetic acid (EDTA) and Lithium Heparin tubes, respectively, and measured or processed within 6 hours after blood collection. Serum and plasma samples for batch analysis of soluble markers were collected in BD Vacutainer® Serum or Lithium Heparin tubes and stored after centrifugation (2000 x g for 10 minutes) at ‑80°C.

Leukocyte cell differentiation, immunophenotyping, ex vivo cytokine secretion, as well as immunoglobulin and albumin determination in serum were carried out in an accredited diagnostic laboratory (Labor Berlin GmbH, Charité/Vivantes, Berlin Germany). The measurements of serum IL 10, cortisol, and transcortin were performed as a batch analysis at the Department of Experimental Neurology at Charité-Universitätsmedizin Berlin, Germany, using commercially available immunoassays of the same lots.

Leucocyte cell differentiation was performed using the XE-5000 Case Manager hematology analyser (Sysmex, Norderstedt, Germany), the Sysmex XN-2000 hematology analyser (Sysmex Canada, Mississauga, Ontario, Canada), and the XE-5000 Case Manager hematology analyser (Sysmex Suisse AG, Horgen, Switzerland) in certified clinical diagnostic laboratories in Berlin, Zurich and Toronto, respectively. Lymphocyte subpopulations were quantified using a ten-color Navios flow cytometer in conjuction with Navios Software (Beckman Coulter). Used fluorescence labelled monoclonal anti-human antibodies from mice (all from Beckman Coulter, Krefeld, Germany) were CD3 Allophycocyanine-Alexa Fluor 750 (APC-A750, clone UCHT1; catalog number A94680), CD4 energy coupled dye (ECD, clone SCFI12T4D11; 6604727), CD8 APC (clone B9.11; IM2469), CD14 Fluorescein isothiocyanate (FITC, clone RMO52; IM0645U), CD16 Phycoerythrine (PE, clone 3G8; A07766), CD19 PE-Cy5.5 (clone J3-119; B49211), CD45RA Pacific-Blue (PB, clone J33; A74763), CD56 PE (clone N901; A07788).

The expression of monocytic Human Leukocyte Antigen-DR (mHLA-DR) was determined by flow cytometry using a highly standardised quantitative assay (BD Quantibrite™, HLA-DR/Monocyte reagent, BD Biosciences, San Jose, CA, USA) according to the standardised working procedures established for clinical diagnosis of immunosuppression. This method was approved in an inter-laboratory study^3^ and is presented more comprehensively in the study protocol publication.^2^

For the evaluation of functional T cell immunity, the secretion of cytokines after ex vivo T cell stimulation with concanavalin A (IFN-γ, TNF‑a, IL‑2, IL‑4, IL‑5, IL‑10) or staphylococcal enterotoxin B (IL‑17A) was quantified. For concanavalin A assays, 200 µl of heparinised whole blood was incubated with 50 µl of concanavalin A (1 mg/ml, Sigma Aldrich) and 750 µl of RPMI 1640 (Biochrom) for 24 hours (37°C, 5% CO_2_). For stimulation with staphylococcal enterotoxin B, 250 µl of heparinised whole blood was incubated with 50 µl staphylococcal enterotoxin B (5 µg/ml, Sigma Aldrich) and 200 µl of RPMI 1640 (Biochrom) for 24 hours (37°C, 5% CO_2_). After 24 hours of incubation, obtained supernatants (1000 x g for 5 min) were stored at ‑80°C until analysis. IL-17A was quantified using the Human IL-17 Quantikine ELISA (R&D Systems, Wiesbaden, Germany). All other cytokines in cell culture supernatants were measured using the Human Th1/Th2 Cytokine Cytometric Bead Array (CBA) (BD Biosciences, Heidelberg, Germany).

Serum immunoglobulin (Ig) and albumin concentrations were determined in vitro by turbidimetric assays using Tina-quant® (Roche, Rotkreuz, Switzerland) at Labor Berlin – Charité Vivantes GmbH (Berlin, Germany). IL-10 serum concentration was measured by using U-PLEX Biomarker Group 1 Immunoassays (#K15067L‑2, MSD, Rockville, Maryland, USA). Serum transcortin also referred as corticosteroid binding globulin (CBG) concertation was determined by the immunoassay CBG ELISA from IBL Hamburg (#BV52021). All assays were conducted according to manufacturer protocols. The free calculated cortisol concentration was derived by using serum levels of cortisol, transcortin, and albumin according to Dorin et al.^4^

Clinically established cell type ratios i.e., neutrophil/lymphocyte ratio, CD4/CD8 ratio, IFN-γ/IL4 ratio, were calculated based on blood cell differentials, lymphocyte subpopulations or cytokines release assays of ex vivo mitogen‑stimulated T-cells, respectively.

# **Supplementary Tables**

## **Supplementary Table S1**

|  | **COaT-SCI study** | **SCIentinel study** |
| --- | --- | --- |
| Sample size | 296 | 70 |
| Study design | Retrospective | Prospective, with published study protocol^2^ |
| Centre | Single centred (ukb) | - Multi centred - Most patients (n=48, 69%) were treated in ukb |
| Recruitment period | October 2010 until December 2017 | August 2011 until October 2014 |
| Key inclusion and exclusion criteria | - Traumatic SCI | - Traumatic SCI - Spine surgery - No immune-relevant comorbidities - No neurological pre-morbid conditions - No life‑threatening multiple injuries - No serious traumatic brain injury - No treatment with high-dose Methylprednisolone |
| Data capture | Data available from clinical records   - Baseline demographics and clinical characteristics - Time of infections (UTI, PI) and occurrence of pressure ulcers | Predefined, and applied consistently criteria   - Baseline demographics and clinical characteristics - Time of infections (UTI, PI) - Longitudinal examinations, measurement of immune parameters, and specimen collection within five time frames: <31 hours, 31–96 hours, 5–9 days, 11–28 days, and 8–12 weeks |
| Definition of pressure ulcers‡ | Any observed pressure ulcers were considered, independent of location or grade. | Pressure ulcers status were recorded at each visit. Based on the result that there was no relevant association of pressure ulcers and UTI was found in the COaT-SCI data and, in addition, some information of pressure ulcers was missing in the SCIentinel study, pressure ulcers were not included in the analysis.  Due to the visit-dependent assessment of pressure ulcers, missing in Table 2 was defined as: no information on pressure ulcer at any visit. |
| Observation period (time since injury) | To focus on observation in primary hospitalisation and early inpatient rehabilitation phases, the maximum observation period was set at approx. 17 weeks (116 days). This limit represents the 75^th^ percentile of observation time of patients who did not have a work-related accident and undergo for insurance reasons rehabilitation in a different centre. Because discharge may be handled differently at other centres, the criteria for discharging ukb patients to rehabilitation can be found in the supplementary material of the paper by Liebscher et al.^5^ | From acute primary hospitalisation to early inpatient rehabilitation. Administrative end of observation at approximately 10 weeks. |

**Supplementary Table S1: Comparison of both data sources regarding study design and variable definitions.** ‡Pressure ulcer classification grades 1 to 4 are based on the European Pressure Ulcer Advisory Panel (EPUAP) and the National Pressure Ulcer Advisory Panel (NPUAP)^6^. Abbreviations: PI = pulmonary infection, SCI = spinal cord injury, ukb = Unfallkrankenhaus Berlin (level-1 trauma centre Berlin), UTI = urinary tract infection.

## **Supplementary Table S2**

|  | Variable |  | HR (95% CI) |
| --- | --- | --- | --- |
| Constant HR over time | Sex | male vs. female | 0.81 (0.57 - 1.14) |
|  | NLI | high vs. low | 0.91 (0.67 - 1.22) |
|  | Pressure ulcer | first vs. no  second vs. no | 1.12 (0.76 - 1.64)  0.79 (0.38 - 1.67) |
|  | CCI | per one unit increase | 0.99 (0.91 - 1.08) |
|  | Age | 40 vs. 30 years  60 vs. 50 years  80 vs. 70 years | 1.07 (1.02 - 1.11)  1.12 (1.04 - 1.21)  1.18 (1.06 - 1.31) |
| Time-varying HR | AIS | A vs. BCD at 14d  A vs. BCD at 30d  A vs. BCD at 60d | 0.98 (0.65 - 1.47)  1.14 (0.84 - 1.55)  1.52 (1.03 - 2.24) |
|  | PI | first vs. no at 14d  first vs. no at 30d  first vs. no at 60d  second vs. no at 30d  second vs. no at 60d  third vs. no at 60d | 0.56 (0.29 - 1.08)  0.95 (0.65 - 1.40)  1.04 (0.69 - 1.56)  0.16 (0.02 - 1.25)  0.17 (0.02 - 1.39)  0.01 (<0.01 - 1.05) |
|  | Accompanying injury | yes vs. no at 14d  yes vs. no at 30d  yes vs. no at 60d | 0.64 (0.43 - 0.97)  0.79 (0.58 - 1.08)  1.18 (0.76 - 1.84) |

**Supplementary Table S2. HR estimates in numerical form presented in Fig. 2 of the Cox regression model investigating associations to first UTI in the COaT-SCI study (n=295 SCI patients).** For categorical variables, the latter category is reference. Because of the non-linear relationship, examples are shown for defined ages, and due to time-varying associations, examples are shown for defined time points after SCI. PI and pressure ulcer are time-varying variables that changed status from “no” to “first”, “second”, and “third” PI or pressure ulcer at the time of the respective event. The NLI categorisation into high (C1-T4) and low (T5-S4/5) was chosen to account for disturbed signaling between the brain and the greater splanchnic nerve originating from spinal segments T5-T9. Abbreviations: CCI = Charlson Comorbidity Index, CI = confidence interval, d = day, HR = hazard ratio, NLI = neurological level of injury, PI = pulmonary infection, S = sacral vertebra, SCI = spinal cord injury, T = thoracic vertebra, UTI = urinary tract infection.

## **Supplementary Table S3**

| Follow-up time point (days) | Concordance summary | Patients at risk for first UTI |
| --- | --- | --- |
| 14 | 0.66 | 250 |
| 21 | 0.64 | 214 |
| 42 | 0.61 | 118 |
| 84 | 0.61 | 35 |

**Supplementary Table S3. Discriminatory ability (risk classification performance) of the applied Cox regression model (first UTI) in the COaT-SCI study (n=295 SCI patients).** The concordance summaries (a weighted average of the time specific AUC)^7^ at 14, 21, 42, and 84 days after SCI are shown. Abbreviations: AUC = Area Under the receiver operating characteristic Curve, SCI = spinal cord injury, UTI = urinary tract infection.

## **Supplementary Table S4**

|  | Variable |  | HR (95% CI) |
| --- | --- | --- | --- |
| Basic model | Sex | male vs. female | 0.33 (0.14 - 0.75) |
|  | NLI | high vs. low | 0.40 (0.18 - 0.91) |
|  | Age | per decade | 1.12 (0.90 - 1.39) |
|  | Accompanying injury | yes vs. no | 0.53 (0.26 - 1.08) |
| Extended model | PI | first vs. no at 10d  first vs. no at 20d  first vs. no at 30d  first vs. no at 40d | 0.86 (0.26 - 2.86)  1.30 (0.50 - 3.35)  1.96 (0.85 - 4.53)  2.97 (1.19 - 7.43) |

**Supplementary Table S4. HR estimates in numerical form presented in Fig. 3 of the basic and the extended Cox regression model investigating associations to first UTI in the SCIentinel study (n=70 SCI patients).** The basic model is stratified for PI and the extended model allows time-varying association between PI and UTI. For categorical variables, the latter category is reference. Due to time-varying associations in the extended model, examples for PI are shown for defined time points after SCI. PI is a time-varying variable that changes status from “no” to “first” PI at the visit or, in case of infection between two visits, at the subsequent visit. The NLI categorisation into high (C1-T4) and low (T5-S4/5) was chosen to account for disturbed signaling between the brain and the greater splanchnic nerve originating from spinal segments T5-T9. Abbreviations: C = cervical vertebra, CI = confidence interval, d = day, HR = hazard ratio, NLI = neurological level of injury, PI = pulmonary infection, S = sacral vertebra, SCI = spinal cord injury, T = thoracic vertebra, UTI = urinary tract infection.

## **Supplementary Table S5**

|  | Model /  Candidate biomarker | Concordance summary  at follow-up time point | | | Patients included / at risk for first UTI at day | HR (95% CI) of  candidate biomarker |
| --- | --- | --- | --- | --- | --- | --- |
|  |  | 14 days | 21 days | 42 days | Included / 14 / 21 / 42 |  |
| - | Basic model ^a^ | 0.69 | 0.68 | 0.65 | 70 / 48 / 44 / 26 | - |
| A | ln (leucocytes/nl ×1000) ^a^ | 0.72 | 0.70 | 0.67 | 68 / 45 / 41 / 24 | 1.34 (0.51 - 3.50) |
| A | ln (neutrophils/nl ×1000) ^a^ | 0.73 | 0.70 | 0.68 | 68 / 45 / 41 / 24 | 1.27 (0.56 - 2.90) |
| A | ln (lymphocytes/nl ×1000) ^a^ | 0.73 | 0.71 | 0.69 | 68 / 45 / 41 / 24 | 0.59 (0.21 - 1.63) |
| A | ln (monocytes/nl ×1000) ^a^ | 0.71 | 0.69 | 0.66 | 67 / 45 / 41 / 24 | 0.67 (0.33 - 1.37) |
| A | ln (NLR) ^b^ | 0.53 | 0.53 | 0.53 | 68 / 45 / 41 / 24 | 2.61 (1.18 - 5.79);  0.86 (0.25 - 2.95) § |
| B | ln (NK cells/nl ×1000) ^a^ | 0.74 | 0.72 | 0.70 | 64 / 43 / 39 / 23 | 0.56 (0.31 - 1.02) |
| B | ln (B cells/nl ×1000) ^a^ | 0.71 | 0.69 | 0.67 | 64 / 43 / 39 / 23 | 1.11 (0.61 - 2.03) |
| B | ln (T cells/nl ×1000) ^a^ | 0.72 | 0.71 | 0.70 | 64 / 43 / 39 / 23 | 0.43 (0.20 - 0.95) |
| B | ln (CD4 T cells/nl ×1000) ^a^ | 0.70 | 0.69 | 0.67 | 64 / 43 / 39 / 23 | 0.85 (0.40 - 1.79) |
| B | ln (CD8 T cells/nl ×1000) ^a^ | 0.78 | 0.76 | 0.74 | 64 / 43 / 39 / 23 | 0.34 (0.18 - 0.65) |
| B | ln (CD4 / CD8 ratio) ^a^ | 0.78 | 0.75 | 0.72 | 64 / 43 / 39 / 23 | 2.66 (1.36 - 5.17) |
| C | ln (TNF-a pg/ml) ^a^ | 0.74 | 0.72 | 0.69 | 63 / 41 / 37 / 21 | 0.79 (0.62 - 1.01) |
| C | ln (IFN-y pg/ml) ^a^ | 0.75 | 0.72 | 0.70 | 63 / 41 / 37 / 21 | 0.64 (0.40 - 1.04) |
| C | ln (IL-2 pg/ml) ^a^ | 0.72 | 0.70 | 0.68 | 63 / 41 / 37 / 21 | 1.15 (0.79 - 1.69) |
| C | ln (IL-4 pg/ml) ^a^ | 0.71 | 0.69 | 0.67 | 63 / 41 / 37 / 21 | 1.56 (0.74 - 3.27) |
| C | ln (IL-5 pg/ml) ^a^ | 0.72 | 0.71 | 0.69 | 63 / 41 / 37 / 21 | 1.59 (0.99 - 2.58) |
| C | ln (IL-10 pg/ml) ^a^ | 0.71 | 0.69 | 0.67 | 63 / 41 / 37 / 21 | 1.07 (0.71 - 1.60) |
| C | ln (IL-17A pg/ml) ^a^ | 0.72 | 0.70 | 0.68 | 53 / 38 / 35 / 19 | 1.13 (0.89 - 1.45) |
| C | ln (IFN-γ / IL-4 ratio) ^a^ | 0.75 | 0.73 | 0.70 | 63 / 41 / 37 / 21 | 0.53 (0.33 - 0.83) |
| D | ln (IgM g/l ×1000) ^a^ | 0.70 | 0.69 | 0.66 | 68 / 45 / 41 / 24 | 0.65 (0.38 - 1.09) |
| D | ln (IgG g/l ×1000) ^a^ | 0.66 | 0.65 | 0.64 | 68 / 45 / 41 / 24 | 0.26 (0.09 - 0.77) |
| D | ln (IgA g/l ×1000) ^b^ | 0.54 | 0.54 | 0.54 | 68 / 45 / 41 / 24 | 0.83 (0.35 - 1.97);  0.20 (0.06 - 0.74) § |
| E | ln (mHLA-DR Ab/cell) ^c^ | 0.77 | 0.76 | 0.74 | 64 / 43 / 39 / 23 | 0.39 (0.14 - 1.06);  2.27 (0.90 - 5.75) § |
| E | ln (IL-10 serum pg/ml) ^b^ | 0.75 | 0.74 | 0.71 | 68 / 45 / 41 / 24 | 1.11 (0.90 - 1.37);  0.76 (0.61 - 0.94) § |
| E | albumin/10 g/l ^a^ | 0.71 | 0.69 | 0.66 | 68 / 45 / 41 / 24 | 0.97 (0.49 - 1.90) |
| E | transcortin/10 µg/ml ^a^ | 0.71 | 0.69 | 0.66 | 68 / 45 / 41 / 24 | 0.90 (0.61 - 1.32) |
| E | ln (free calc. cortisol nM) ^b^ | 0.50 | 0.50 | 0.51 | 68 / 45 / 41 / 24 | 0.93 (0.51 - 1.69);  0.32 (0.13 - 0.77) § |

**Supplementary Table S5.** **Discriminatory ability of Cox regression models (first UTI) in the SCIentinel study**. The discriminatory ability (predictive performance) is described by the concordance summary (a weighted average of the time specific AUC)^7^ at 14, 21, and 42 days after SCI. The basic model consists of the variables sex, neurological level of injury (high vs. low), AIS (A vs. BCD), accompanying injury (yes vs. no), age, and the time-varying status of PI. Each candidate biomarker was used in a separate regression model with covariates mentioned in the basic model. Superscripts indicate the type of Cox regression model used (a, stratification for PI; b, stratification for PI and interaction between strata and biomarker; c, interaction between PI and biomarker). Derived HR estimates of candidate biomarkers, illustrated in Fig. 2, are presented in numbers. When an interaction term was included (PI strata x immune parameter; PI x immune parameter), two estimates are shown. The first gives the estimator for patients who not yet experienced PI, and the second (indicated with §) when PI has already occurred. Candidate biomarkers are assigned to peripheral blood immune cell counts (A, B), ex vivo mitogenic stimulated T cell cytokine release (C), serum immunoglobulin concentrations (D), and further immune and immune associated parameters (E). Abbreviations: Ab = antibody, AUC = Area Under the receiver operating characteristic Curve, calc. = calculated, CD = cluster of differentiation, CI = confidence interval, HR = hazard ratio, IFN‑γ = interferon gamma, Ig = immunoglobulin, IL = interleukin, ln = natural logarithm, mHLA‑DR = monocytic Human Leukocyte Antigen-DR, NK cells = natural killer cells, NLR = neutrophil to lymphocyte ratio, PI = pulmonary infection, SCI = spinal cord injury, TNF-a = tumor necrosis factor alpha, UTI = urinary tract infection.

# **Supplementary Figures**

## **Supplementary Figure S1**


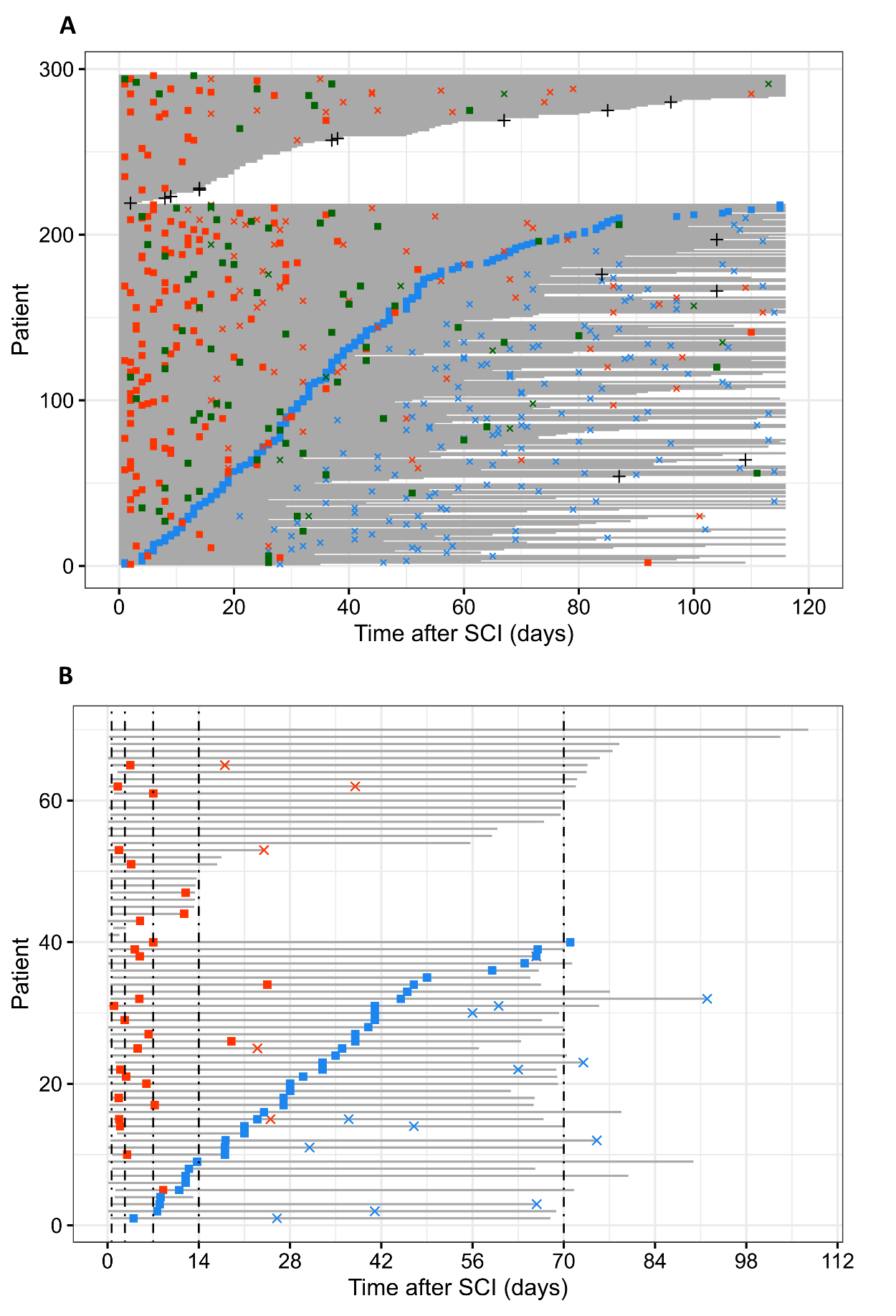


**Supplementary Figure S1. Onset and frequency of observed infections**. (A) COaT-SCI study (B) SCIentinel study (B). Each horizontal line (grey) indicates the follow-up time of each patient. Squares represent first infection or pressure ulcer, Xs the repeated occurrence of these, and a black cross indicates death during acute stay. PI is colored red, UTI blue and pressure ulcer green. The exact time of pressure ulcer was only captured in the COaT-SCI study. Vertical dashed lines depict the planned visit times of the SCIentinel study. Patients were ordered by time of first UTI and then by time of follow-up. Abbreviations: PI = pulmonary infection, SCI = spinal cord injury, UTI = urinary tract infection.

## **Supplementary Figure S2**


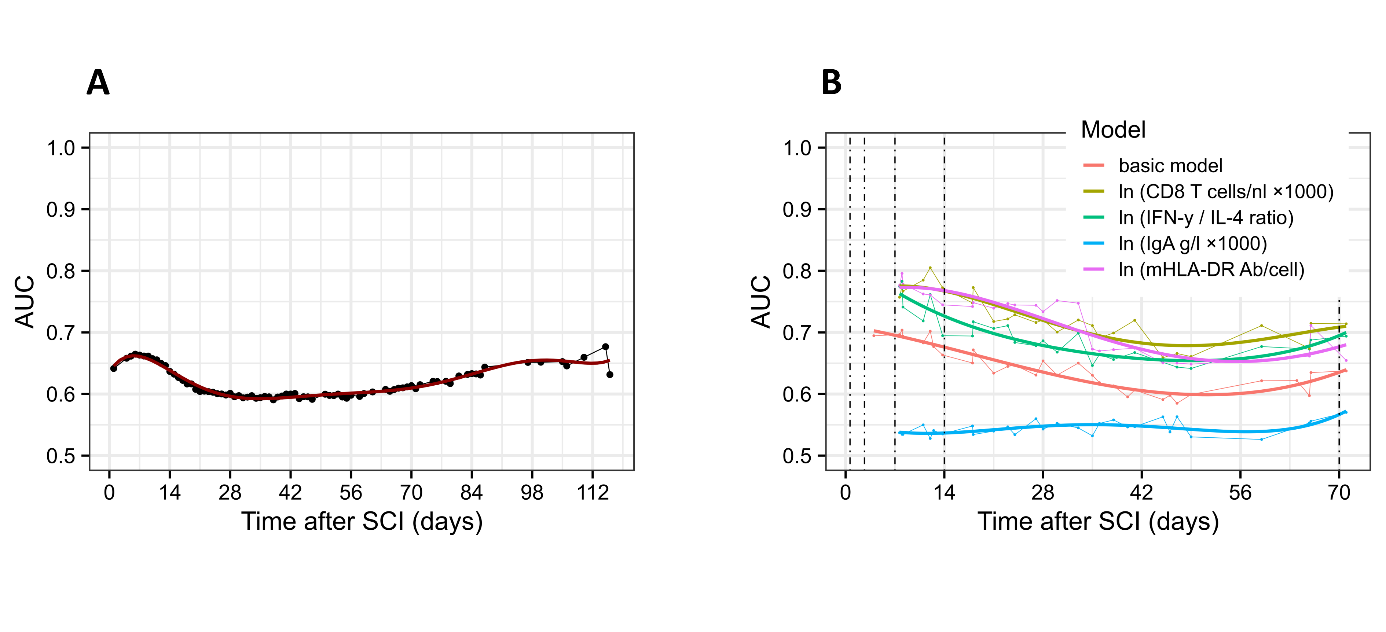


**Supplementary Figure S2. Incident/dynamic area under the receiver operating characteristic curve (i/d AUC).** i/d AUC^7^ at observed UTI event times using the linear predictor of the applied Cox regression models in (A) the COaT-SCI study and (B) the SCIentinel study. Higher AUC values (ranging from 0.5-1.0) indicate better discriminatory performance to distinguish between patients with high versus low UTI risk at each time point. Vertical dashed lines represent the planned visit time points of the SCIentinel study. Abbreviations: Ab = antibody, AUC = area under the curve, CD = cluster of differentiation, IFN‑γ = interferon gamma, Ig = immunoglobulin, ln = natural logarithm, SCI = spinal cord injury.

## **Supplementary Figure S3**


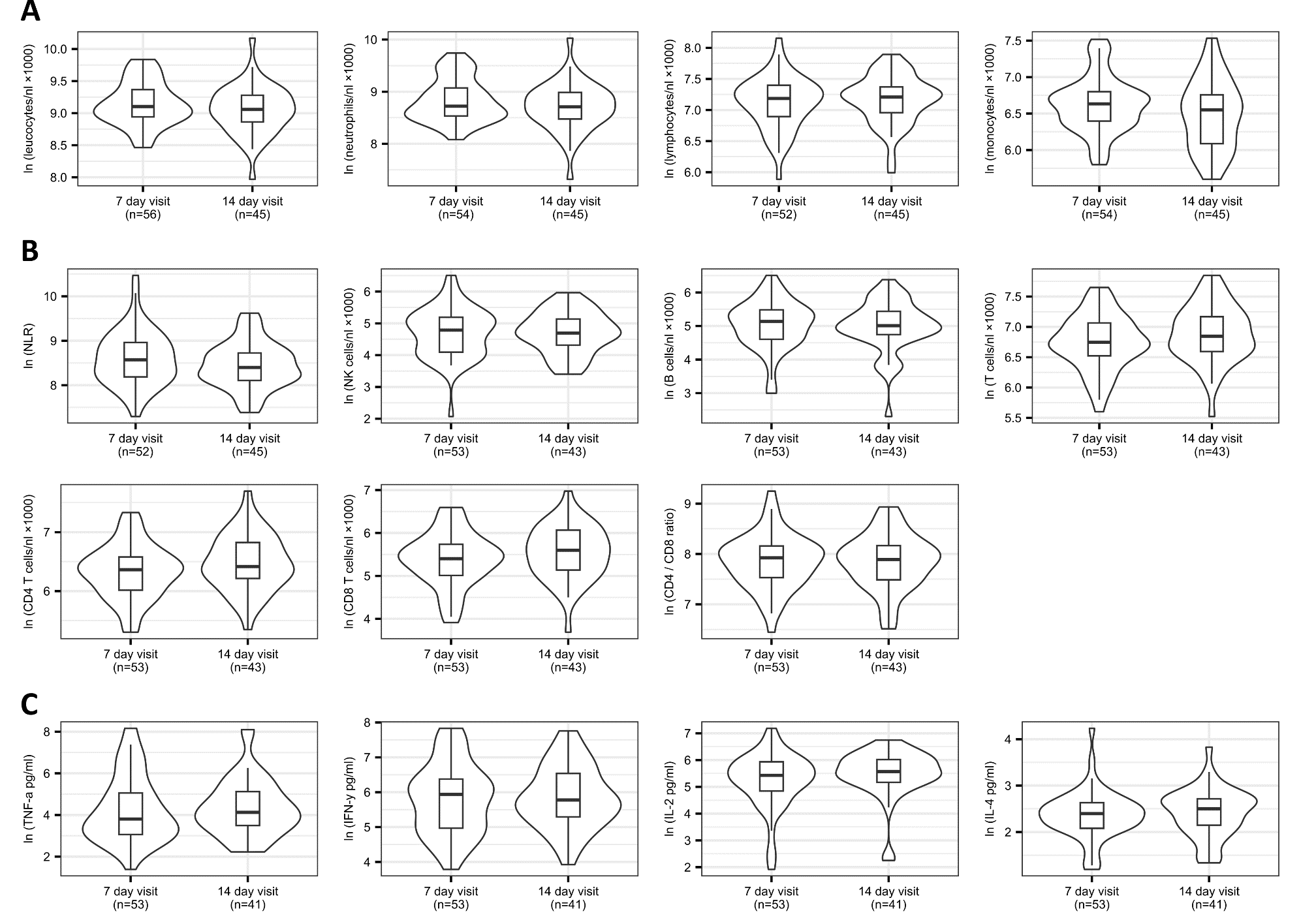


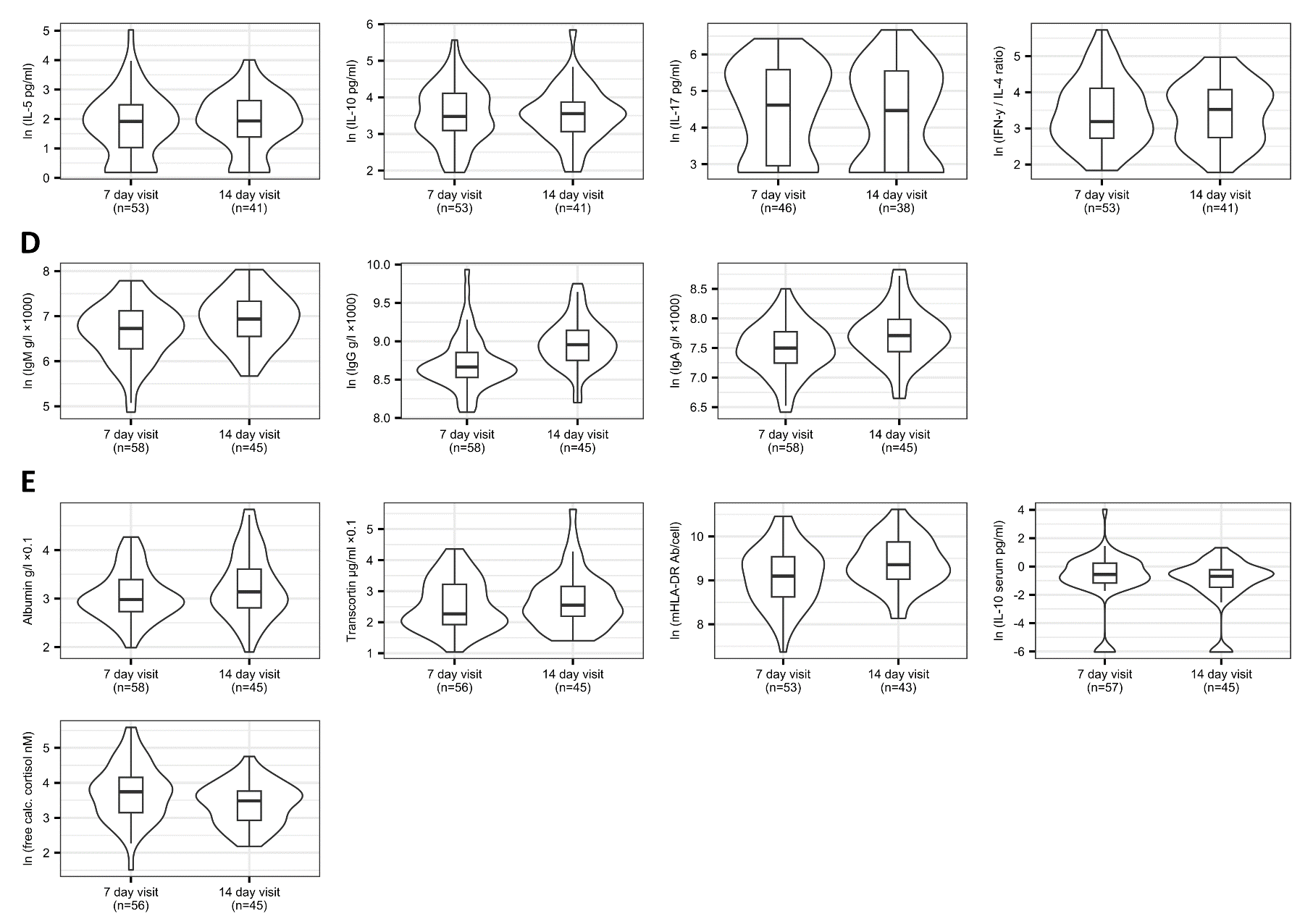


**Supplementary Figure S3. Distributions of transformed immune and immune-related laboratory measurements at the 7-day and 14-day visits of the SCIentinel study.** The visits shown are the closest visits prior to the occurrence of the first UTI. Only data from patients that were at risk for the first UTI were used. The distributions are presented as violin plots and box plots (lower and upper hinges correspond to the first and third quartiles; whiskers extend from the lower or upper hinge to the lowest or highest value no further than 1.5 times the interquartile range; outlying points are displayed only in the violin plots). Shown parameters are peripheral blood immune cell counts (A, B), cytokine release of ex vivo mitogen-stimulated T cells in whole blood (C), serum immunoglobulin (Ig) concentrations (D), and further immune and immune-related parameters (E). Abbreviations: Ab = antibody, calc. = calculated, CD = cluster of differentiation, IFN-γ = interferon gamma, Ig = immunoglobulin, IL = interleukin, ln = natural logarithm, mHLA-DR = monocytic Human Leukocyte Antigen-DR, NK cells = natural killer cells, NLR = neutrophil to lymphocyte ratio, TNF-a = tumor necrosis factor alpha, UTI = urinary tract infection.

## **Supplementary Figure S4**

**
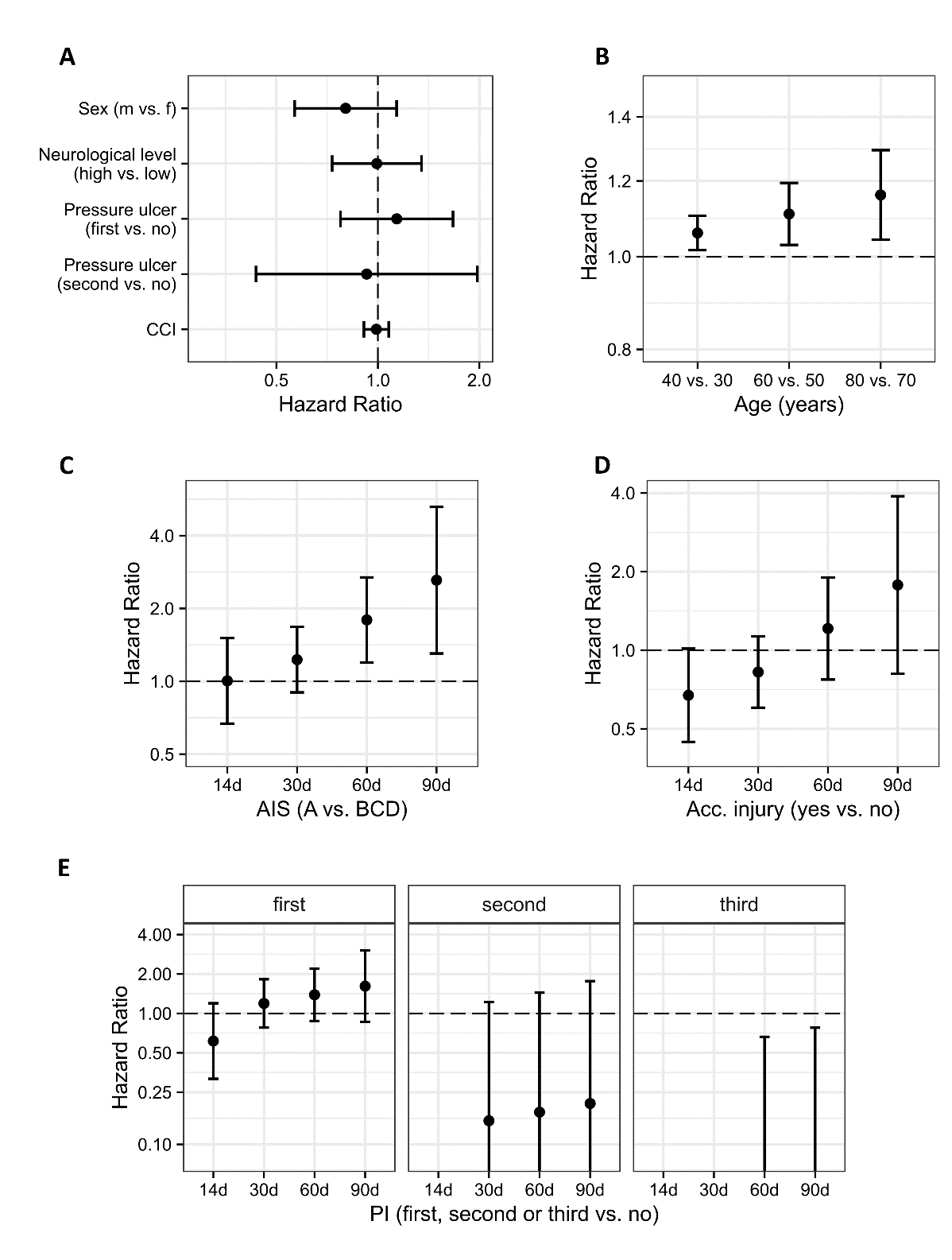
**

**Supplementary Figure S4. Association of clinical risk factors with first UTI in the COaT-SCI study when additionally adjusted for length of first stay in ICU.** The analyses were done as described before (Figure 2), except that the estimates were additionally adjusted for the length of stay in the ICU (summed over time, incorporated as a time-dependent variable), which corresponds to the minimum duration of indwelling catheter use. All associations remained very similar. The HR (95% CI) for the length of first stay in the ICU was 0.98 (0.97 - 1.00) per day and points in the same direction as the HR for the duration of initial urethral indwelling catheterisation in patients with acute traumatic SCI reported elsewhere for the time to first UTI.^8^ Hazard Ratios and 95% CI for first UTI for variables with constant hazard (A), non-linear hazard (B), and time varying hazard over time (C-E). The applied Cox regression model includes extensions of time varying covariates (PI and pressure ulcer) and time varying hazards (PI, AIS, and accompanying injury). All estimates were adjusted for the other shown variables in this figure (A-E). Abbreviations: acc. injury = accompanying injury, AIS = American Spinal Injury Association Impairment Scale, AUC = area under the receiver operating characteristic curve, CCI = Charlson Comorbidity Index at baseline, f = female, m = male, NLI = neurological level of injury, PI = pulmonary infection, SCI = spinal cord injury, UTI = urinary tract infection.

## **Supplementary Figure S5**

**
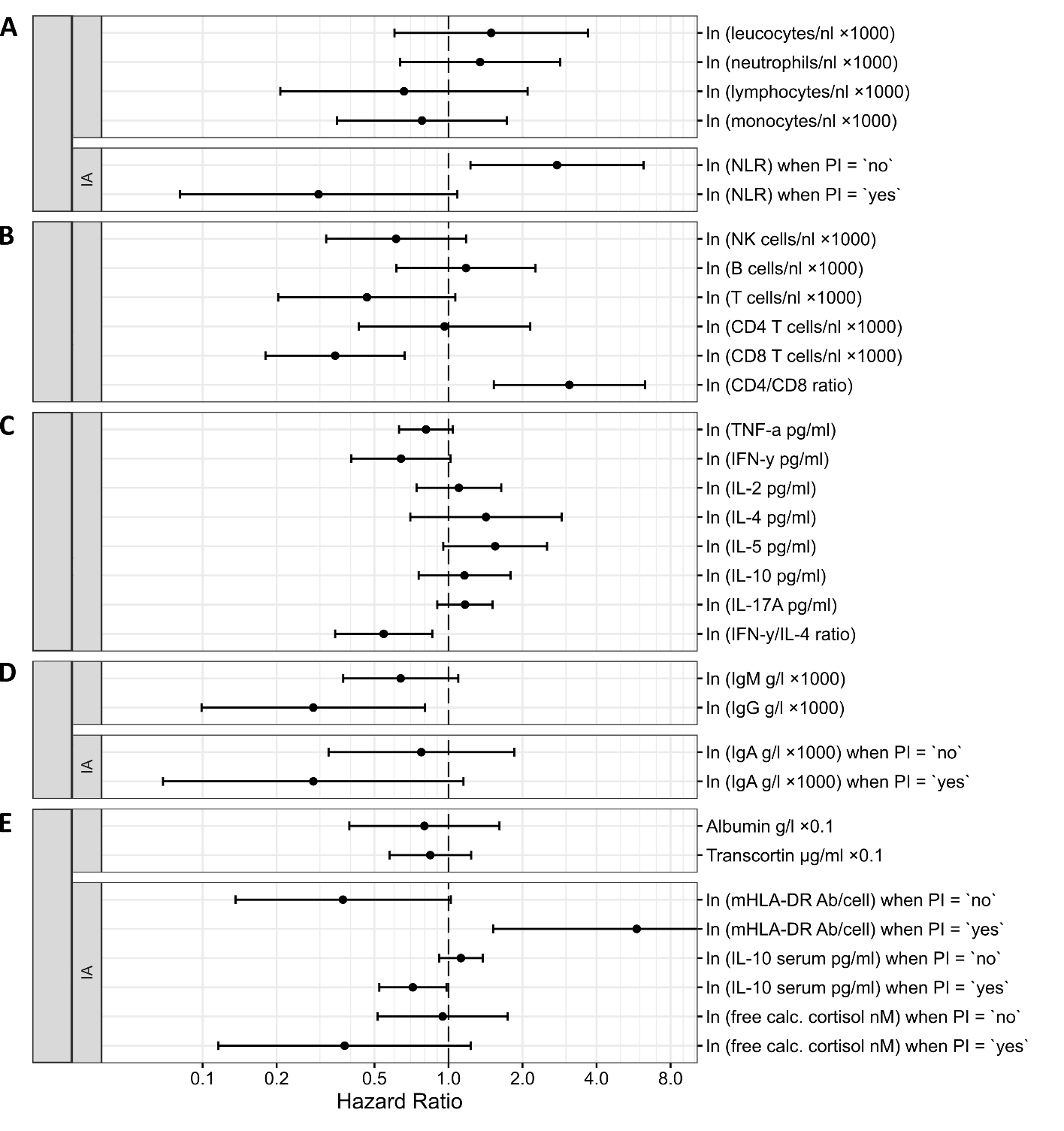
**

**Supplementary Figure S5. Immunological candidate markers for UTI risk in the SCIentinel study when additionally adjusted for a surrogate for the method of catheterisation.** The analyses were done as described before (Figure 4), except that estimates were additionally adjusted for the length of first stay in the ICU (summed up to the corresponding visit as a time-dependent variable), which corresponds to the minimum duration of indwelling catheter use. Associations of biomarkers with first UTI were very similar. Differences to previous results were observed only for NLR and mHLA DR and only for the post-PI estimates, indicating a more pronounced interaction for both, but the meaning of the results remained. The HR (95% CI) for the length of first stay in the ICU was 0.94 (0.86 - 1.03) per day using the basic model without biomarker and points in the same direction as the HR for the duration of initial urethral indwelling catheterisation in patients with acute traumatic SCI reported elsewhere for the time to first UTI.^8^ Associations of peripheral blood immune cell counts (A-B), cytokine release of ex vivo mitogen-stimulated T cells in whole blood (C), serum immunoglobulin concentrations (D), and further immune and immune related parameters (E) to first UTI using Cox regression models with time varying covariates in patients of the SCIentinel study. Each potential biomarker was evaluated in a separate Cox model. Subpanels are labelled IA when PI × immune parameter is present, meaning the association to UTI was different after PI occurred. The time-varying status of PI changed from `no` to `yes` when PI was detected before or at the corresponding visit. Shown estimates were adjusted for age, sex, neurological level of injury, severity of injury, the presence of accompanying injury, and, when no PI interaction term was included, for PI. Abbreviations: Ab = antibody, calc. = calculated, CD = cluster of differentiation, IA = interaction, IFN-γ = interferon gamma, Ig = immunoglobulin, IL = interleukin, ln = natural logarithm, mHLA-DR = monocytic Human Leukocyte Antigen-DR, NK cells = natural killer cells, NLR = neutrophil to lymphocyte ratio, PI = pulmonary infection, TNF-a = tumor necrosis factor alpha, UTI = urinary tract infection.

# **Supplementary References**

1 Kopp, M. A. *et al.* The spinal cord injury-induced immune deficiency syndrome: results of the SCIentinel study. *Brain* **146**, 3500–3512 (2023). <https://doi.org/10.1093/brain/awad092>

2 Kopp, M. A. *et al.* The SCIentinel study--prospective multicenter study to define the spinal cord injury-induced immune depression syndrome (SCI-IDS)--study protocol and interim feasibility data. *BMC Neurol* **13**, 168 (2013). <https://doi.org/10.1186/1471-2377-13-168>

3 Döcke, W. D. *et al.* Monitoring temporary immunodepression by flow cytometric measurement of monocytic HLA-DR expression: a multicenter standardized study. *Clin Chem* **51**, 2341–2347 (2005). <https://doi.org/10.1373/clinchem.2005.052639>

4 Dorin, R. I. *et al.* Validation of a simple method of estimating plasma free cortisol: role of cortisol binding to albumin. *Clin Biochem* **42**, 64–71 (2009). <https://doi.org/10.1016/j.clinbiochem.2008.09.115>

5 Liebscher, T. *et al.* Cervical Spine Injuries with Acute Traumatic Spinal Cord Injury: Spinal Surgery Adverse Events and Their Association with Neurological and Functional Outcome. *Spine (Phila Pa 1976)* **47**, E16–e26 (2022). <https://doi.org/10.1097/brs.0000000000004124>

6 National Pressure Ulcer Advisory Panel, European Pressure Ulcer Advisory Panel & Pan Pacific Pressure Injury Alliance. Prevention and Treatment of Pressure Ulcers: Quick Reference Guide. *Cambridge Media: Osborne Park, Western Australia* (2014).

7 Heagerty, P. J. & Zheng, Y. Survival model predictive accuracy and ROC curves. *Biometrics* **61**, 92–105 (2005). <https://doi.org/10.1111/j.0006-341X.2005.030814.x>

8 Goodes, L. M. *et al.* Early urinary tract infection after spinal cord injury: a retrospective inpatient cohort study. *Spinal Cord* **58**, 25–34 (2020). <https://doi.org/10.1038/s41393-019-0337-6>
